# Cellular and molecular architecture of submucosal glands in wild-type and cystic fibrosis pigs

**DOI:** 10.1073/pnas.2119759119

**Published:** 2022-01-19

**Authors:** Wenjie Yu, Thomas O. Moninger, Andrew L. Thurman, Yuliang Xie, Akansha Jain, Keyan Zarei, Linda S. Powers, Alejandro A. Pezzulo, David A. Stoltz, Michael J. Welsh

**Affiliations:** ^a^Department of Internal Medicine, Pappajohn Biomedical Institute, Roy J. and Lucille A. Carver College of Medicine, University of Iowa, Iowa City, IA 52242;; ^b^Department of Molecular Physiology and Biophysics, Pappajohn Biomedical Institute, Roy J. and Lucille A. Carver College of Medicine, University of Iowa, Iowa City, IA 52242;; ^c^Department of Biomedical Engineering, University of Iowa, Iowa City, IA 52242;; ^d^HHMI, University of Iowa, Iowa City, IA 52242

**Keywords:** lung, epithelia, cystic fibrosis, mucus, submucosal gland

## Abstract

Submucosal glands (SMGs) protect lungs but can also contribute to disease. For example, in cystic fibrosis (CF), SMGs produce abnormal mucus that disrupts mucociliary transport. CF is an ion transport disease, yet knowledge of the ion transporters expressed by SMG acini, which produce mucus, and SMG ducts that carry it to the airway lumen is limited. Therefore, we isolated SMGs from newborn pigs and used single-cell messenger RNA sequencing, immunohistochemistry, and in situ hybridization to identify cell types, gene expression, and spatial distribution. Cell types and transcript levels were the same in non-CF and CF SMGs, suggesting that loss of epithelial anion secretion rather than an intrinsic cell defect causes CF mucus abnormalities. Gene signatures of acinar mucous and acinar serous cells revealed specialized functions in producing mucins and antimicrobials, respectively. However, surprisingly, these two cell types expressed the same ion transporters and neurohumoral receptors, suggesting the importance of balancing mucin and liquid secretion to produce optimal mucus properties. SMG duct cell transcripts suggest that they secrete HCO_3_^−^ and Cl^−^, and thus have some similarity to pancreatic ducts that are also defective in CF. These and additional findings suggest the functions of the SMG acinus and duct and provide a baseline for understanding how environmental and genetic challenges impact their contribution to lung disease.

## Significance

Submucosal glands secrete antimicrobial proteins and mucus into the airway lumen to protect the lung by killing inhaled and aspirated pathogens and clearing them from the lung. They can also contribute to several lung diseases, including the genetic disease cystic fibrosis. To better understand their structure and function, we isolated and studied submucosal glands from newborn pigs. Normal and cystic fibrosis submucosal glands were similar, suggesting that disease is due to loss of anion secretion rather than an intrinsic cell defect. By identifying submucosal gland cell types and the messenger RNA they express, the data aid understanding of submucosal gland function and provide a baseline for learning how environmental and genetic challenges contribute to lung disease.

A major challenge for lungs is the continuous inhalation and frequent aspiration of microorganisms and foreign material. To meet this challenge, prevent infection, and clear particulates, the conducting airways have evolved a variety of defense mechanisms (1–7). In cartilaginous airways of humans, pigs, and other large mammals, submucosal glands (SMGs) are essential components of defense (1, 8–13). SMGs secrete liquid and most of the mucus in large airways; the secreted mucus binds microorganisms and particulate material, and ciliary beating propels the mucus out of the lung in the process of mucociliary clearance (1, 2, 5, 8–10, 13, 14). SMGs also produce abundant antimicrobial peptides and proteins that prevent infection by killing bacteria and inhibiting viruses (1, 11, 15–18).

However, SMGs can also contribute to the pathophysiology of lung disease. In cystic fibrosis (CF), SMGs produce abnormally elastic mucus that disrupts mucociliary transport (14, 19–24). In chronic obstructive pulmonary disease and asthma, SMGs hypertrophy and produce mucus that obstructs the airways (1, 2).

Despite their importance in maintaining healthy lungs and in contributing to lung disease, understanding SMG cellular architecture and molecular composition lags far behind understanding of surface epithelia that line the airways. Knowledge is limited in part because mice, the most commonly used laboratory mammal, lack SMGs except in the most proximal part of the airway (25, 26). SMGs isolated from humans are limited in availability and there is uncertainty about changes due to both apparent and unrecognized environmental and disease-related factors. SMGs are also relatively small and inaccessible, and while studies of electrolyte transport have been informative, they are limited to isolated serous cells (27, 28). Moreover, culture models have not replicated the three-dimensional structure of SMGs, which are organized into two regions, acini and ducts (29, 30). The acinus contains epithelial mucous cells that produce mucin and serous cells that secrete ions, liquid, and antimicrobials (1, 9–13, 16, 27, 28, 31, 32). Mucus flows from the acini to the airway surface through a series of ducts that merge to form a single terminal duct.

To better understand the cellular and molecular bases of SMG function, we studied the porcine lung. The size, anatomy, biochemistry, and physiology of the pig lung closely resemble those of the human lung (33). They have a similar amount and distribution of SMGs (26, 33, 34), and porcine SMGs have been used as a model in many physiological studies (21, 22, 35). Like humans, pigs have SMGs at birth, and by studying newborn pigs it is possible to avoid secondary manifestations of environmental exposures and disease. Pigs can also model human disease, including CF lung disease (36–39). *CFTR*^null^ and *CFTR*^ΔF508^ pigs develop airway infection, inflammation, and mucus occlusion much as seen in people with CF (19, 40, 41). Moreover, their SMGs exhibit duct obstruction and produce abnormally acidic mucus with increased elasticity that impairs mucociliary transport (18, 20, 22, 23, 42).

Here we investigated the single-cell transcriptome and cellular organization of SMGs from the newborn pig. We also tested non-CF and CF SMGs to learn if genotype-dependent differences in gene expression might explain differences in function.

## Results

### Analysis of Sequenced SMG Cells Required an Improved Porcine Reference Genome.

**Fig. 1.**
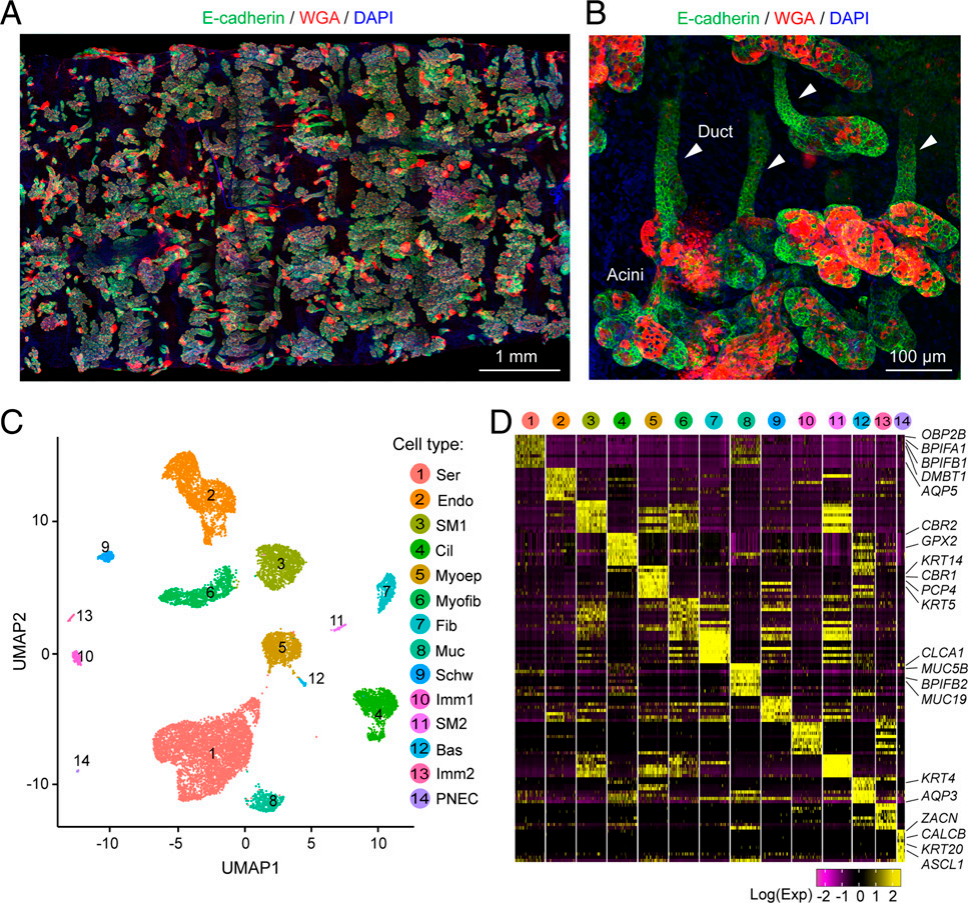
scRNA-seq of newborn pig SMGs reveals multiple cell types. (*A*) Representative immunofluorescent image of SMGs after tissue was removed from the cartilage. Images were taken from the basolateral side of the tissue and stitched together. Staining is E-cadherin (green, an epithelial marker), WGA (red, a lectin interacting with MUC5B), and DAPI (blue). Structures with E-cadherin and WGA double-positive signals are SMGs. (*B*) Representative image of individual SMGs stained with E-cadherin and WGA in whole-mount SMG tissues. Acini are at the bottom and ducts are indicated with arrowheads. (*C*) UMAP of 14 cell clusters in scRNA-seq of SMG and surrounding tissues. Bas; basal cells; Cil, ciliated cells; Endo, endothelial cells; Fib, fibroblasts; Imm, immune cells; Muc, mucous cells; Myoep, myoepithelial cells; Myofib, myofibroblasts; Schw, schwann cells; Ser, serous cells; SM, smooth muscle cells. (*D*) Heatmap of top 10 cell-type signature genes. Representative markers from epithelial clusters are shown. Colors indicated scaled single-cell gene expression (maximum 200 cells were shown) at natural log scale.

We isolated SMGs from trachea of four wild-type and four *CFTR*^−/−^ newborn pigs. We removed the trachea, opened it along its ventral surface, scraped away most surface epithelial cells, and peeled the remaining tissue layer away from the cartilage (*SI Appendix*, Fig. S1). Fig. 1 *A* and *B* shows the SMGs and their structure composed of acini and ducts. The density and depth of SMGs would seem to fit well with the conclusion that the volume of glands is ∼50 times that of surface goblet cells (1). In addition, this layer included surrounding connective tissue and a few residual surface epithelial cells. We then manually dissected and isolated individual SMGs for single-cell dissociation.

We profiled 14,561 single-cells using droplet-based 3′ single-cell RNA sequencing (scRNA-seq). We found that reads for many genes were not well aligned to reference genes, primarily because of the lack of 3′ untranslated regions (UTR) of those reference genes (*SI Appendix*, Fig. S2 *A–D*). To improve accuracy of the reference genome annotation, we manually checked sequencing read alignments for >6,000 genes that are expressed in human, pig, and mouse airways. We then modified the original RefSeq annotation by: 1) adding or elongating the 3′ UTR, 2) reducing the overlap of the UTR region of two genes that have the same orientation, 3) updating gene names, and 4) adding new genes based on other pig genome databases. In addition, all modifications had to refer to existing annotations among University of California, Santa Cruz (UCSC), RefSeq, Ensembl, and GenBank messenger RNA (mRNA) databases. We modified annotations for 241 genes, ∼3.7% of the total genes that we checked (*SI Appendix*, Fig. S2 *E–G* and Dataset S1). We generated a new pig reference genome and generated new matrix datasets based on it.

### Wild-Type and *CFTR*^−/−^ SMGs Did Not Substantially Differ by Transcripts.

We subjected all filtered wild-type and *CFTR*^−/−^ datasets to integrative analysis with the Seurat R toolkit (*SI Appendix*, Fig. S3). We identified 14 major cell clusters (6 epithelial and 8 nonepithelial), which we visualized with two-dimensional uniform manifold approximation and projection (UMAP) (Fig. 1*C* and *SI Appendix*, Fig. S4*A*). Clusters contained as many as 5,469 and as few as 25 cells. We confirmed that cells were evenly distributed inside individual clusters regardless of genotype and batch (*SI Appendix*, Fig. S4*B*). We identified marker genes for each cluster based on unsupervised analysis (Fig. 1*D*, *SI Appendix*, Fig. S4*C*, and Dataset S2) and assigned each cluster to an existing cell-type in airways according to markers that are well known for a cell type (*SI Appendix*, Fig. S4*D*).

Cell-types included those classically reported for SMGs: serous, mucous, and myoepithelial cells (Fig. 1 *C* and *D*) (1, 32, 43, 44). We also identified basal and ciliated cells, which could potentially have derived from the airway surface. Three rare epithelial cell types have been reported in airway surface epithelia: tuft cells, ionocytes, and pulmonary neuroendocrine cells (PNECs) (43–46). We detected PNECs and ionocytes but no tuft cells. In addition, there were eight nonepithelial cell types: endothelial cells, fibroblasts, myofibroblasts, two types of smooth muscle cells, two types of immune cells, and Schwann cells (*SI Appendix*, Fig. S4). These nonepithelial cells create the microenvironment for SMGs between the airway surface and the cartilage rings.

**Fig. 2.**
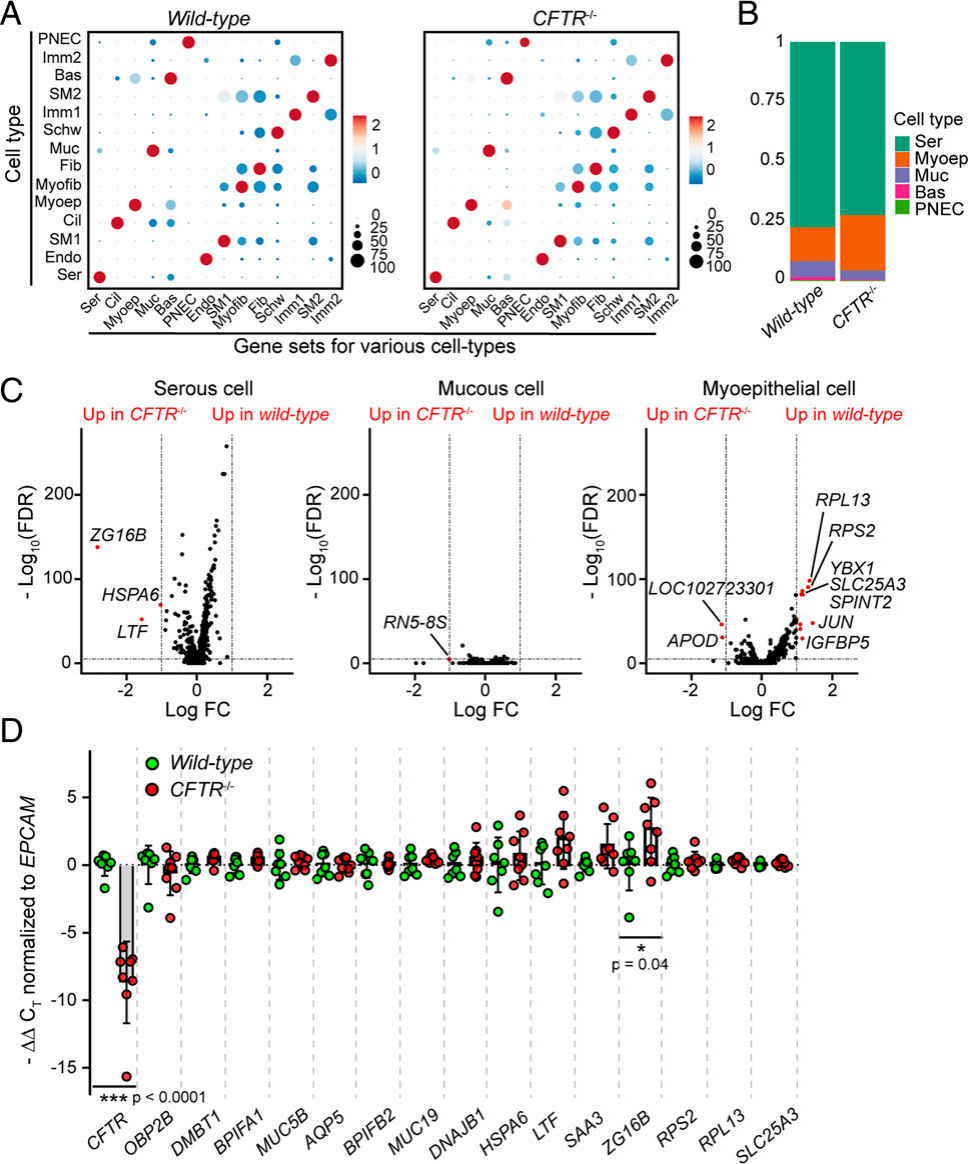
Wild-type and *CFTR*^−/−^ SMGs exhibit similar gene expression profiles. (*A*) Dot plots of selected marker genes that were enriched in each cell type separated by genotype. *y* axis, cell types; *x* axis, mean expression of indicated marker-gene sets. The size of the dot indicates the proportion of cells that were positive for the gene sets in specific cell-types. Color of dots indicates the expression of the gene sets (scaled by row) at natural log scale. (*B*) Bar graph of epithelial cell-type proportion in wild-type and *CFTR*^−/−^ SMG. (*C*) Volcano plots of differentially expressed genes in serous, mucous, and myoepithelial cells between wild-type and *CFTR*^−/−^ SMGs. LogFC indicates fold-change between wild-type and *CFTR*^−/−^ at natural log scale. Dashed lines indicate the cutoffs: genes that were 1 LogFC higher or lower with false-discovery rate (FDR) < 10^−5^ in wild-type are highlighted in red. (*D*) qRT-PCR analysis of serous and mucous cell markers and differentially expressed genes between wild-type and *CFTR*^−/−^ from *C*. CT values for each gene were normalized to the CT value of *EPCAM*. Primers for *CFTR* targeted the deleted exon in *CFTR*^−/−^ pigs (36). Each dot represents a pig; *P* values were generated using unpaired Student’s *t* test.

We compared data from wild-type and *CFTR*^−/−^ pigs. Marker-gene expression did not differ between cells from wild-type and *CFTR*^−/−^ SMGs (Fig. 2*A*). Moreover, the proportion of epithelial cell-types did not significantly differ by genotype (Fig. 2*B*), although there were more epithelial cells in the wild-type dataset (*SI Appendix*, Fig. S4*B*). In addition, within SMG-specific cell types, only a few genes showed differential expression between wild-type and *CFTR*^−/−^ (Fig. 2*C* and Dataset S3). qRT-PCR of multiple genes identified by scRNA-seq revealed only one differentially expressed gene (*zymogen granule protein 16B*, *P* < 0.05) between the two genotypes (Fig. 2*D*). These results suggest that wild-type and *CFTR*^−/−^ SMGs have a similar cellular and molecular composition. In subsequent analyses, we combined data from all eight pigs.

### Distinct Cell Types Suggest an Architecture for SMG Acini.

SMGs were organized with multiple acini connecting to a single duct (Fig. 1*B* and *SI Appendix*, Fig. S5). To reconstruct the spatial distribution of cell types and the architecture of SMGs, we used immunostaining and single-molecule fluorescence in situ hybridization (smFISH) with representative markers.

**Fig. 3.**
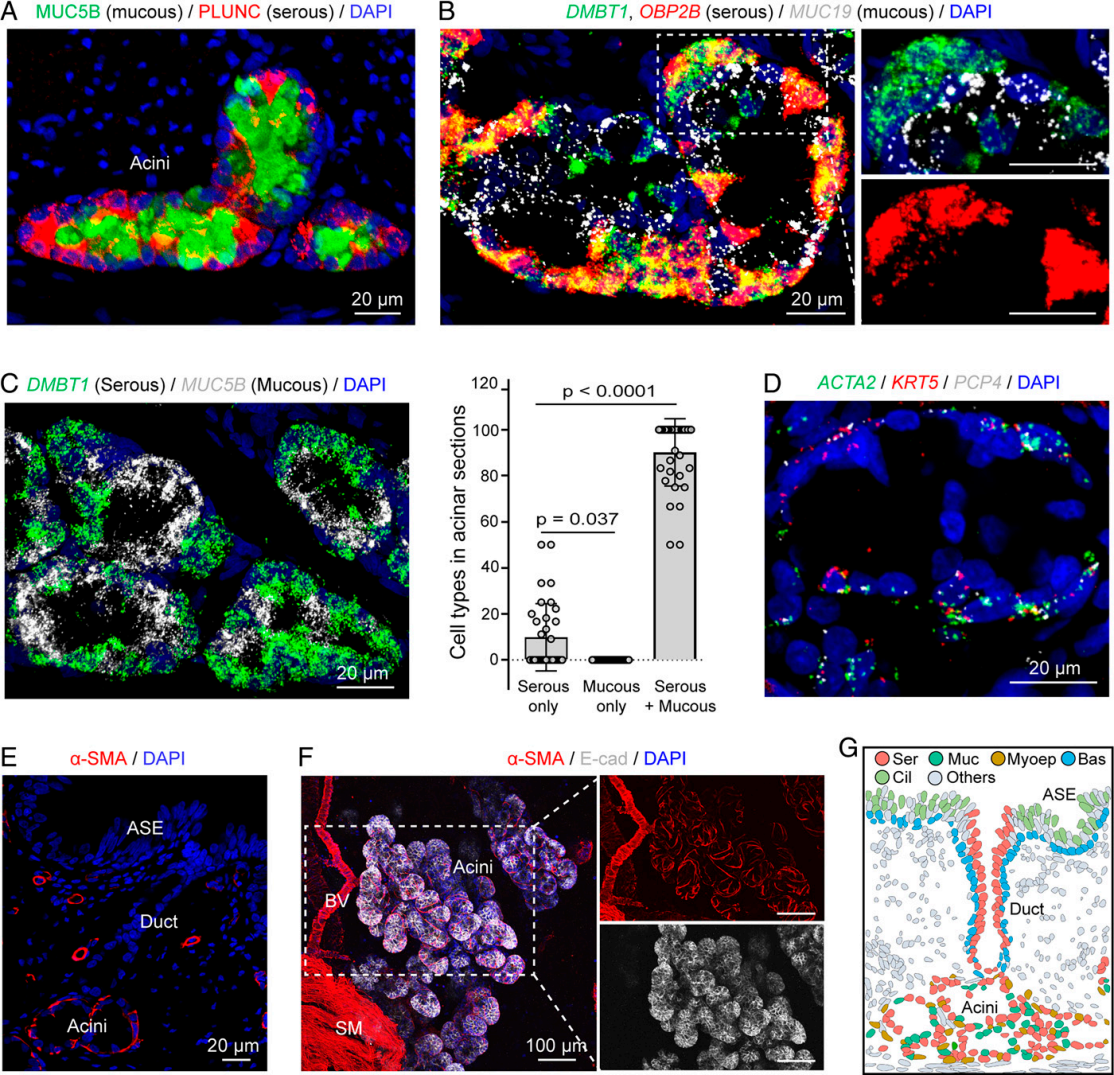
SMG acini contain a mix of serous and mucous cells covered with myoepithelial cells. (*A*) Representative image of SMG immunostained for of PLUNC (BPIFA1) and MUC5B. (*B*) Representative image of smFISH of serous cell (*DMBT1, OBP2B*) and mucous cell (*MUC19*) markers in SMG acinus. (*C*) Analysis of cellular composition of SMG acinus. (*Left*) Representative image of smFISH of *DMBT1* (serous cell marker) and *MUC5B* (mucous cell marker) in SMG acini. (*Right*) Summary of acinar sections containing pure serous cell population, pure mucous cell population, or a mix of these two cell types. Each data point represents an image. Statistical comparison was based on averaged values of multiple images from a pig; *n* = 4 pigs, unpaired Student’s *t* test. (*D*) Representative images of smFISH of myoepithelial cell (*ACTA2*, *KRT5, PCP4*) markers in the acinar region of SMGs. (*E* and *F*) Representative images of immunostaining of αSMA (protein name for *ACTA2*) in SMG tissue sections and whole tissue. αSMA was localized in the acini, not the duct of SMGs. BV, blood vessel; E-cad, e-cadherin, labeled the entire population of epithelial cells in SMGs; SM, smooth muscle. (*G*) Model of spatial distribution of epithelial cell types from scRNA-seq. Each dot represents a nucleus from a real tissue section. Colors for epithelial cell types are matched to cell types in Fig. 1*C*. ASE, airway surface epithelia.

Acini were composed primarily of three cell types: serous, mucous, and myoepithelial cells. Serous cells were positive for *BPIFA1* (PLUNC), *DMBT1*, *OBP2B*, and mucous cells were positive for *MUC5B* and *MUC19* (Fig. 3 *A–C*). In general, serous cells were mixed with mucous cells throughout most of the acinus. Occasionally serous cells formed a cluster at the very distal part of an acinus, but we did not find areas with only mucous cells (Fig. 3*C*, *SI Appendix*, Fig. S5, and Video S1). This organization differs from a report of human SMGs, in which serous cells were located in the distal portion of an acinus branch, and mucous cells clustered in the proximal portion of acinus branches (29).

Myoepithelial cells were positive for *ACTA2*, *KRT5*, and *PCP4*, a new marker for myoepithelial cells (Figs. 1*D* and 3*D* and *SI Appendix*, Fig. S6). We found that myoepithelial cells wrapped around the acinus of SMGs (Fig. 3 *E* and *F*). Previous reports suggest that myoepithelial cells of airway SMGs facilitate gland contraction and mucus secretion in response to neural signals (1, 47). Consistent with that suggestion, their shape and gene-expression signature suggested contractile features.

These findings identify and localize cell types in the porcine SMG acinus (Fig. 3*G*).

### Acinar Serous and Mucous Cells Both Express Transcripts for Host Defense.

**Fig. 4.**
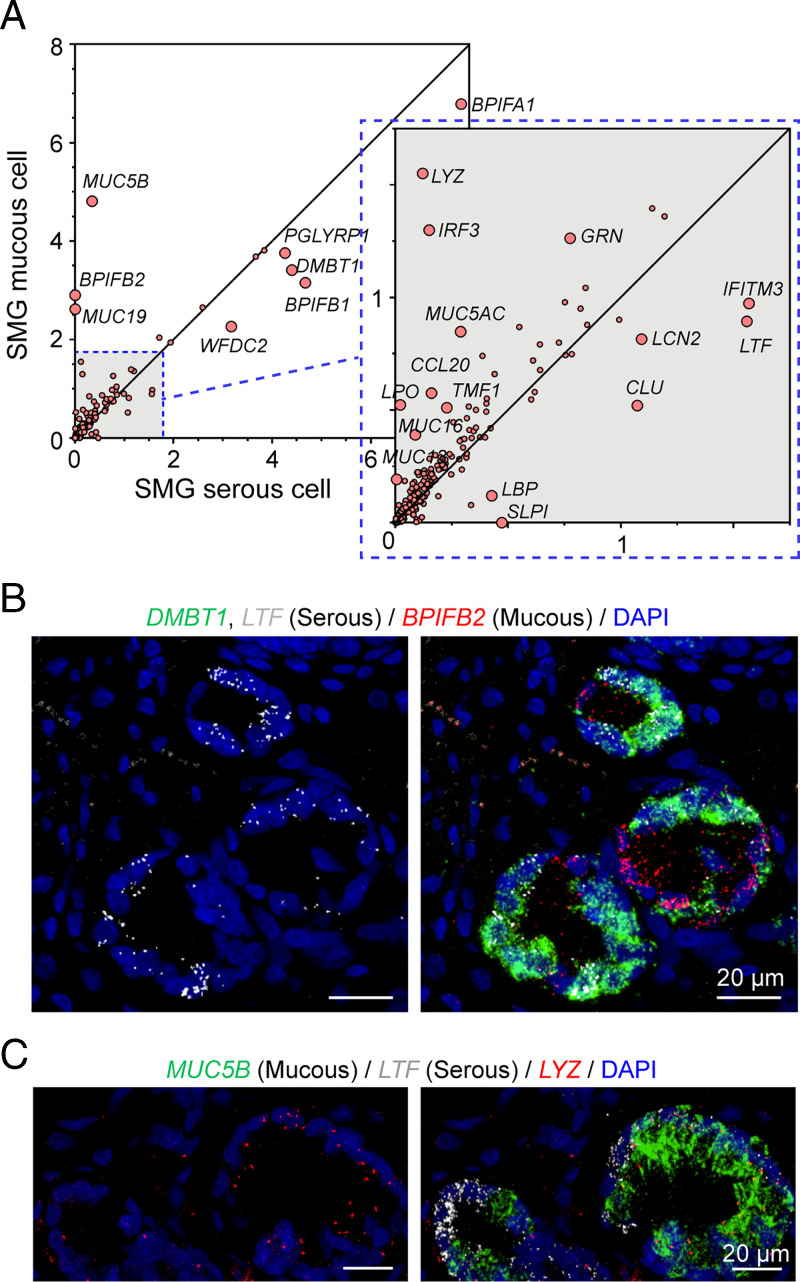
Acinar serous and mucous cells have distinct expression patterns of host defense genes. (*A*) Scatter plot of mean expression of host defense genes in mucous and serous cells at natural log scale. The dots for labeled genes are enlarged for better visualization. (*B*) Representative images of smFISH of *LTF*, *DMBT1*, and *BPIFB2* in SMGs. *LTF* colocalized with *DMBT1*. (*C*) Representative images of smFISH of *LYZ* in SMGs. *LYZ* colocalized with MUC5B signals.

To further predict distinct and shared functions of SMG serous and mucous cells in host defense, we compared their levels of transcripts. We curated a set of host defense-related genes (Dataset S4), and found that serous and mucous cells were specialized for some host defense-related genes. For example, mucous cells expressed *MUC5B* and *MUC19* mucin genes at much higher levels than serous cells (Fig. 4*A*). Also as expected, compared to mucous cells, serous cells were enriched for transcripts for several secreted proteins, including secretory leukocyte peptidase inhibitor (SLPI), lactoferrin (LTF), and bactericidal permeability increasing proteins (BPI variants) (Fig. 4 *A* and *B*). However, the distinction was not absolute: mucous cells also expressed some of these host defense genes. As an example, mucous cells preferentially expressed *BPIFB2* (Fig. 4 *A* and *B*). Moreover, an antimicrobial usually ascribed to serous cells, lysozyme (LYZ), unexpectedly showed fourfold greater expression in mucous cells (Fig. 4 *A* and *C*).

### Acinar Serous and Mucous Cells Express Similar Transcript Levels for Transepithelial Ion and Liquid Transport.

**Fig. 5.**
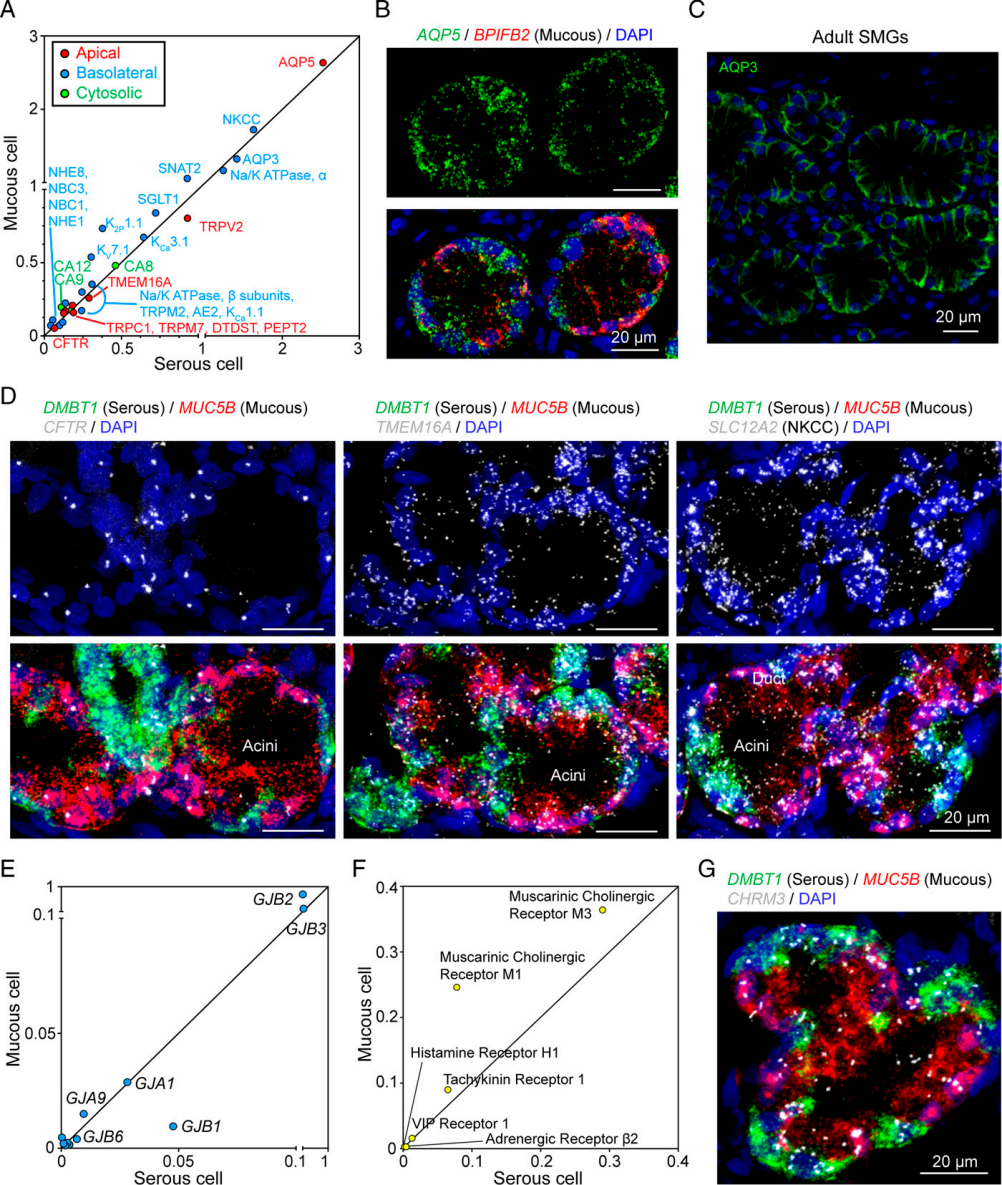
Acinar serous and mucous cells express similar transcripts for ion transporters. (*A*) Scatter plot of mean expression of transepithelial ion transporters in mucous and serous cells at natural log scale. Different colors represent different subcellular localization of indicated transporters. (*B* and *C*) Representative images of aquaporin staining in SMG acini. In *B*, *AQP5* is equally distributed in *BPIFB2*^+^ (mucous) cells and *BPIFB2*^−^ (serous) cells. In *C*, AQP3 protein is localized on the basolateral side of all acinar cells of adult SMGs. (*D*) Representative images of the expression pattern of key ion transporters (CFTR, TMEM16A, NKCC) in the acinus of SMGs. Serous cells (*DMBT1*^+^) are shown in green; mucous cells (*MUC5B*^+^) are shown in red. (*E*) Scatter plot of the expression of gap junction genes in serous and mucous cells. The line across the plot indicates equal expression of genes between serous and mucous cells. (*F*) Scatter plot of mean expression of secretory-related receptors in mucous and serous cells at natural log scale. (*G*) Representative image of smFISH of *CHRM3* (gene for muscarinic cholinergic receptor M3) in SMG acini. *DMBT1* (green) and MUC5B (gray) are markers for serous cells and mucous cells, respectively.

To better understand the potential role of the two acinar cell types in electrolyte and liquid secretion, we also curated a set of genes that encode proteins involved in transepithelial electrolyte and water transport (Dataset S4). We were surprised to find that serous and mucous cells had similar transcript levels for genes involved in anion secretion, including apical anion channels TMEM16A (ANO1) and CFTR; basolateral anion transporters including the Na^+^/K^+^/2Cl^−^ cotransporter (NKCC), the Na^+^/H^+^ exchangers (NHE1 and NHE8), the Na^+^/HCO_3_^−^ cotransporter (NBC1, NBC3), plus carbonic anhydrases (CA9 and CA12) that generate intracellular HCO_3_^−^ (Fig. 5 *A* and *D*). In addition, both cell types expressed similar transcript levels for a basolateral (AQP3) and an apical (AQP5) aquaporin, the Na^+^-K^+^-ATPase, and K^+^ channels (Fig. 5 *A–C* and *SI Appendix*, Fig. S7). Moreover, the cell types contained similar mRNA levels for gap junction genes, suggesting coupling between the two cell types (Fig. 5*E*).

Secretory activity will depend, in part, on plasma membrane receptors that sample neurohumoral signals. We did not identify a receptor that was unique to either serous or mucous cells; both expressed receptors for acetylcholine, substance P, VIP, histamine, and adrenergic agonists (Fig. 5 *F* and *G*).

### SMG Ducts Are Comprised Predominantly of Serous and Basal Cells.

**Fig. 6.**
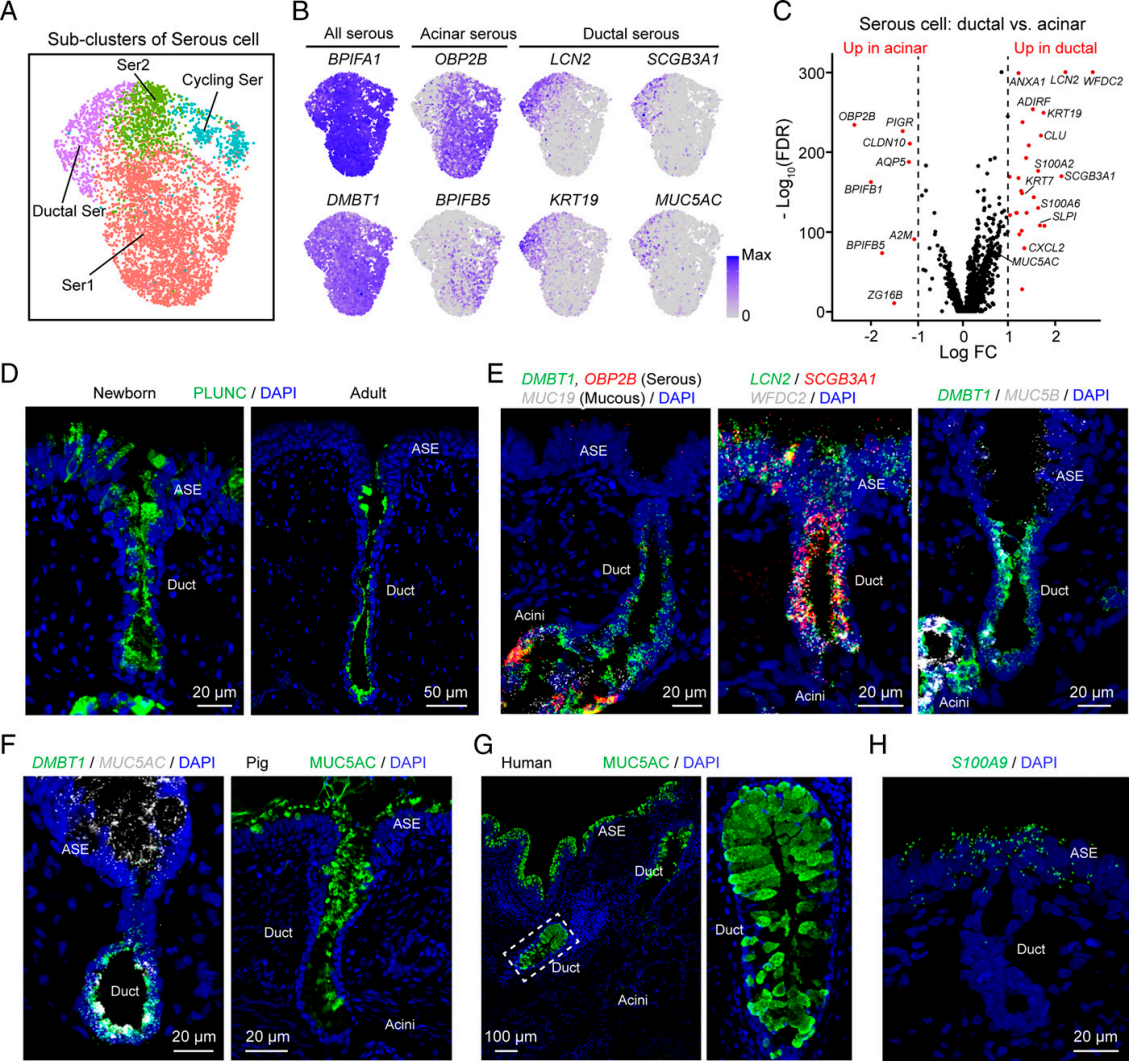
SMG ducts have a unique subcluster of serous cells. (*A*) Subclusters from the UMAP serous-cell cluster in Fig. 1*C*. The serous-cell cluster was further grouped into four subclusters. Subclusters include two types of acinar serous cells (Ser1, Ser2), serous cells in division (Cycling Ser), and ductal serous cells (Ductal Ser). (*B*) Expression pattern of representative markers for acinar serous cells and ductal serous cells in UMAP. (*C*) Volcano plot of genes differentially expressed (red) between ductal serous cells and acinar serous cells (Ser1). (*D*) Representative images of immunostaining of PLUNC (*BPIFA1*) in the duct of newborn (*Left*) and adult (*Right*) SMGs. (*E*) Representative images of smFISH of secretory genes (*DMBT1*, *OBP2B*, *MUC19, LCN2*, *SCGB3A1*, *WFDC2*, *MUC5B*) in the duct of SMGs. (*F*) Representative images of smFISH (*Left*) and immunostaining (*Right*) of MUC5AC in the duct of pig SMGs. (*G*) Representative images of immunostaining of MUC5AC in the duct of human SMGs. (*H*) Representative image of smFISH of *S100A9* (a marker for airway surface secretory cells) in airway surface epithelia and SMGs. The SMG duct and acinus did not express *S100A9*.

We noticed that when we labeled with two serous cell markers, not all *DMBT1*^+^ cells were also positive for *OBP2B* (Fig. 3*B*). Therefore, to assess heterogeneity of serous cells, we reanalyzed the large serous cell cluster and identified four subclusters (Fig. 6 *A* and *B*). A subcluster of ductal serous cells had unique gene expression features (Fig. 6 *C* and *D*, *SI Appendix*, Fig. S8, and Datasets S5 and S6) and localized along the inner layer of SMG ducts. Ductal serous cells expressed markers that were common in other serous cells, such as *DMBT1* and *BPIFA1* (PLUNC), but they did not express some markers typical of acinar serous cells, such as *OBP2B* and *BPIFB5* (Fig. 6 *B–E* and *SI Appendix*, Fig. S8*B*). In addition, ductal serous cells expressed some genes expressed by airway surface secretory cells—such as *LCN2*, *SCGB3A1*, *SLPI*, and *MUC5AC*—but not others, such as *S100A8*, *S100A9*, and *S100A12* (Fig. 6 *B*, *C*, and *E–H*). Thus, ductal serous cells share some features with acinar serous cells and surface secretory cells; however, they differ from both. Consistent with these findings, we detected secretory vesicles in SMG duct cells (*SI Appendix*, Fig. S9). We also localized the basal cells to the outer layer of SMG ducts (*SI Appendix*, Fig. S10), basal cells, and ductal serous cells form the SMG duct epithelia.

Our scRNA-seq data also identified some ciliated cells, and previous studies reported ciliated cells in human SMG ducts (29, 48). However, smFISH and immunostaining for three ciliated cell markers, FOXJ1, TMEM212, and acetylated α-tubulin, localized them exclusively to surface epithelia with none in SMG ducts (*SI Appendix*, Figs. S11 and S12). We also examined airways with immunohistochemical staining for acetylated α-tubulin in older pigs and found that the pseudostratified surface epithelium with ciliated cells sometimes dipped down in toward the SMG duct before the duct developed a cuboidal structure (*SI Appendix*, Fig. S12). However, the cuboidal duct epithelium did not contain ciliated cells in newborn or adult pigs. Because this result contrasts with data from human SMG ducts (29, 48), as a control we reproduced that finding by obtaining human tissue and detecting FOXJ1^+^ ciliated cells in SMG ducts (*SI Appendix*, Fig. S13). Thus, ciliated cells in our scRNA-seq data were from the airway surface and the pseudostratified epithelium at the most proximal portion of the duct, and in contrast to human SMG ducts, the cuboidal epithelia of pig SMG ducts do not contain ciliated cells.

Although ionocytes were rare in the scRNA-seq data, immunostaining for BSND, a marker for ionocytes, revealed ionocytes in SMG ducts (*SI Appendix*, Fig. S14). These results are consistent with previous reports that CFTR-expressing ionocytes are located in the ducts (43, 49).

**Fig. 7.**
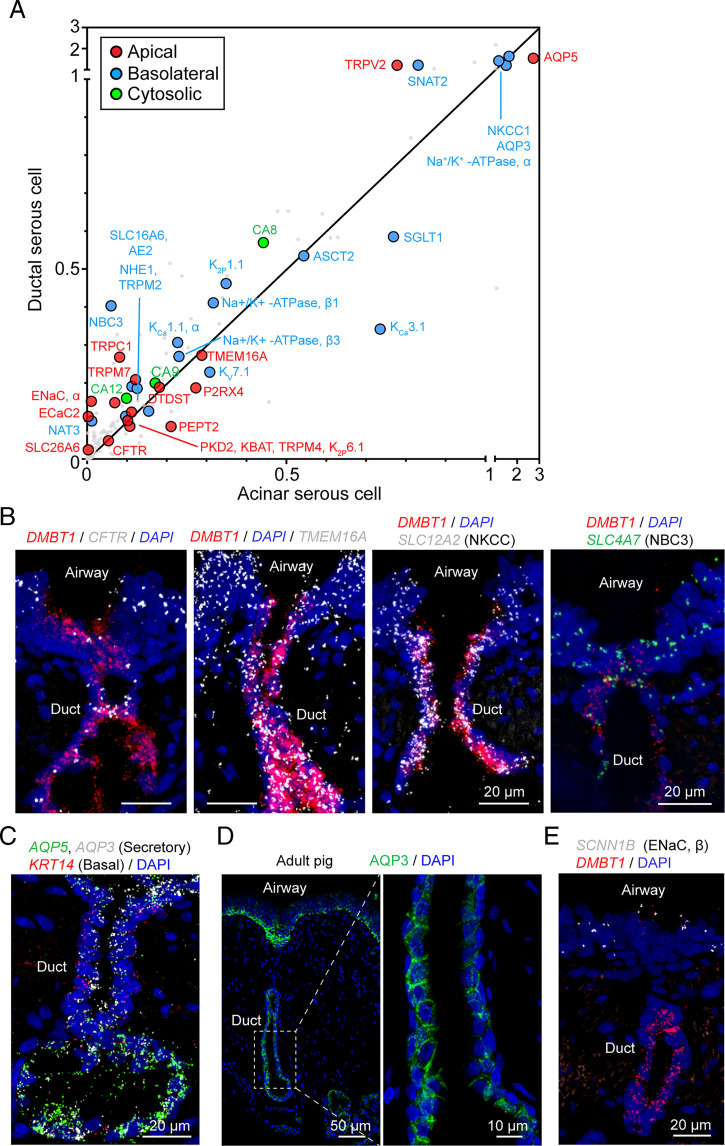
SMG ductal serous cell transcripts indicate an anion secretory function. (*A*) Scatter plot of the mean expression of transepithelial electrolyte transporter genes in ductal and acinar serous cells at natural log scale. Different colors of dots indicate different subcellular localization of the encoded protein. (*B*) Representative images of the expression pattern of key ion transporters (CFTR, TMEM16A, NKCC, NBC3) in the duct of SMGs. Ductal serous cells (*DMBT1*^+^) are shown in red. (*C*) Representative image of smFISH of *AQP3*, *AQP5*, and *KRT14* in tracheal sections. In SMGs, *AQP3* and *AQP5* are expressed in both ductal serous cells and acinar serous cells. *KRT14* is expressed in basal cells of the duct and myoepithelial cells of the acinus. (*D*) Representative images of immunostaining of AQP3 in airway sections of an adult pig. Enlarged image shows that AQP3 is localized at the basolateral region of ductal serous cells. (*E*) Representative images of smFISH of *SCNN1B* (encodes the β-subunit of ENaC) in tracheal sections. SMG duct (*DMBT1*^+^ region, red) was not labeled with *SCNN1B* signal.

To further assess potential contributions to ion and water secretion, we examined the curated set of genes involved in transepithelial electrolyte and water transport (Fig. 7*A* and Dataset S4). Ductal serous cells express channels and transporters that enable transepithelial HCO_3_^−^ and Cl^−^ secretion; these include NKCC, AE2, NBC3 that transport anions across the basolateral membrane, and TMEM16A and CFTR that allow anion exit from the cell across the apical membrane (Fig. 7 *A* and *B*). Also consistent with an epithelial transport function, ductal-serous cells expressed both basolateral (AQP3) and apical (AQP5) aquaporins (Fig. 7 *A*, *C*, and *D*). Expression of these genes encoding transporters involved in anion and liquid secretion were similar in ductal and acinar serous cells. Also consistent with a secretory rather than an absorptive function, we detected transcripts for αENaC but not the other required subunits (Fig. 7 *A* and *E*); ENaC is a key channel for Na^+^ absorption in airway surface epithelia (50).

## Discussion

We studied porcine SMGs at single-cell resolution with RNA sequencing, smFISH, and immunohistochemistry to construct SMG molecular signature and infer function. The data support and expand previous observations and interpretations. However, the results also provide new insight into the molecular architecture and potential cell-type–specific functions.

### Acinar Serous and Mucous Cells Share Mechanisms for Anion and Liquid Secretion.

Substantial functional evidence has assigned anion and liquid secretion to serous cells (1, 27, 28, 51). Consistent with previous evidence that mucous cells can contribute to C1^−^ secretion (1), our findings suggest that mucous cells secrete anions and liquid. We found that mucous cells express the same transcripts at approximately the same levels as serous cells for proteins that mediate anion secretion. These include the apical anion channels CFTR and TMEM16A (52, 53) and the basolateral NKCC and NBC1 and NBC3. Both cell types express cytosolic carbonic anhydrases (CA8, CA12, and CA9) that couple with basolateral NHE8 and NHE1 to generate HCO_3_^−^ for secretion. For water transport, both cell types express apical and basolateral aquaporins (AQP5 and AQP3). In addition, both cell types express gap junction proteins, suggesting that the cells are coupled so that the electrical driving forces across their basolateral and apical membranes may be the same. Moreover, both cell types express a similar group of receptors that may stimulate secretion.

These results suggest that both mucous and serous cells mediate Cl^−^ and HCO_3_^−^ secretion and raise the possibility that the two cell types may make relatively comparable contributions. Consistent with this conclusion, it is reported that substance P causes rapid cell shrinkage, an assay of anion secretions, and at the end of stimulation, both cell types quickly restore their volume (1, 54). We speculate that when SMGs are stimulated, this arrangement of ion transporters, aquaporins, gap junctions, and receptors may serve to ensure a balance between the amount of secreted liquid, the concentration of protein, and the pH in the acinar lumen. Those properties are critical for producing mucus with normal biophysical properties and for ensuring activity of antimicrobial peptides and proteins (18, 20, 22, 55, 56).

### SMG Ducts May Secrete HCO_3_^−^ and MUC5AC.

In addition to providing a conduit, gland ducts often modify their contents. For example, in the sweat gland, ducts absorb Na^+^ and Cl^−^, and in the pancreas, ducts secrete HCO_3_^−^ (57–59). CF disrupts the function of both of these ducts. Whether or how SMG ducts modify mucus flowing to the airway lumen is uncertain. Previous reports about whether SMG ducts express ENaC Na^+^ channel subunits vary (60–62). An extracellular electrophysiological study suggested that porcine SMG ducts absorbed Na^+^ and Cl^−^ and reduced the Na^+^ and C1^−^ concentrations of secreted liquid (63). Another study indicated that SMG ducts do not alter the liquid volume of the duct lumen (64). Moreover, Widdicombe and Wine (1) concluded that ion transport by duct epithelia was very unlikely to alter duct liquid volume given the trivial area of ductal epithelia vs. the area of acinar epithelia.

We found that transcripts for α, β, and γ ENaC Na^+^ channel subunits were absent or of low abundance in duct serous cells, although they may have been below our level of detection. However, duct serous cells expressed multiple genes required for transepithelial anion secretion. These results suggest that porcine SMG ducts have a secretory function, and we speculate that HCO_3_^−^ secretion is the main transport function of the ducts. This hypothesis is also based on the observation that the pH of liquid emerging from SMG ducts is ∼7.5 (22, 55). That pH value puts the HCO_3_^−^ concentration above equilibrium for a basolateral pH of 7.4. In addition, an electrically negative lumen places the HCO_3_^−^ concentration substantially above electrochemical equilibrium. In the pancreatic duct, HCO_3_^−^ secretion is supported by Cl^−^ exit through CFTR channels followed by Cl^−^ reentry in exchange for HCO_3_^−^ exit on the SLC26A6 Cl^−^/HCO_3_^−^ exchanger (65). A similar mechanism may exist in serous duct cells, which also express SLC26A6.

SMGs produce mucus with a MUC5B core coated with MUC5AC (14, 23). Acinar mucous cells produce the MUC5B core, and we speculate that as it flows through the duct, serous cells secrete MUC5AC that coats the MUC5B core. Of course, MUC5AC derived from surface goblet cells could also coat the strands. The reason for this arrangement of mucins is unknown and selective properties of different gel-forming mucins remain uncertain. We wonder if MUC5B might be more important for the elasticity and toughness of mucus strands, and MUC5AC might be more important for adhesiveness and binding microbes and particulate material.

### Acinar Serous and Mucous Cells Have Specialized Protein Secretion Functions.

Previous examinations have revealed mucin production as a major function of acinar mucous cells and antimicrobial protein/peptide production as a major function of acinar serous cells (1, 12, 32). Our results are consistent with those contributions to respiratory host defense. For example, mucous cells expressed higher mRNA levels for the mucins *MUC5B* and *MUC19*, and acinar serous cells expressed higher mRNA levels for antimicrobial proteins lactoferrin, secretory leukocyte peptidase inhibitor, and bactericidal permeability increasing proteins. However, these distinctions were not absolute; for example, mucous cells had more transcripts for the antimicrobial lysozyme than did serous cells, which differs from human SMGs in which serous cells secrete lysozyme (66). Secretion of both mucins and antimicrobial proteins into the same small acinar lumen raises the question of whether the two might interact with antimicrobials decorating mucins, perhaps based on the charge of the negative mucins and the positive antimicrobials. Such an interaction might facilitate delivery or localization of the antimicrobials (67).

### Porcine and Human SMGs May Exhibit Some Differences.

Mucous produced by human SMG acini is reported to empty into an enlarged duct, called a collecting duct, before exiting through a long thin duct to the airway surface (29). Porcine SMGs did not have a well-defined collecting duct. However, a review of SMG structure suggests that the presence of collecting ducts is variable in humans and other species (48). It is also possible that a region resembling a collecting duct may develop in older pigs (68).

In porcine SMG ducts, the pseudostratified surface epithelium with its ciliated cells dips down into the duct for a very short distance and then immediately transitions to duct serous and basal cells lacking cilia. This differs from human SMG ducts, which are ciliated. The presence or absence of ciliated cells may relate to the length of ducts. In pigs, SMGs are localized between the cartilage layer and airway lumen (8). However, in humans, SMGs sometimes invaginate deep beneath the airway lumen, extending between cartilage rings (29). We speculate that beating cilia may be required to pull mucus up the long ducts in humans. In contrast, pressure from mucus and liquid secretion and contractile activity of myoepithelial cells may be sufficient to push mucus up porcine SMG ducts.

Despite the structural differences between human and pig SMGs noted above, in both humans and pigs, CF SMG produce mucus strands having abnormally increased elasticity that prevents normal mucociliary clearance and the ducts are plugged with mucus (19, 20, 22, 23, 42, 56).

### This Study Has Advantages and Limitations.

This study has advantages. First, studying freshly dissected SMGs rather than performing scRNA-seq on cells from full-thickness airway tissue minimizes the necessity of using transcripts to infer a SMG location. Second, studying SMG cells obtained directly from animals avoids the uncertain effects that culturing can have on cell types and transcript profiles. Third, our conclusions are based on a combination of data from scRNA-seq, in situ hybridization, immunohistochemistry, and histology. Fourth, studying SMGs from newborn pigs avoids potential confounding influences of environmental factors, inflammation, infection, and disease.

This study also has limitations. First, we show that porcine and human SMGs have some differences. However, the differences are limited, and lungs and SMGs of pig and human share many features (33). Moreover, the biochemistry, physiology, and anatomy of the porcine lung are similar to those of humans. Second, as is common in scRNA-seq studies, the proportion of various cell types can be biased by differential isolation, and varying dissociation, viability, and lysis that can misrepresent the true proportion of the various cell types. Fortunately, our main conclusions are not dependent on the relative proportions of the various cell types.

### Data from SMG Acini and Ducts Have Implications for CF and Other Airway Diseases.

By studying SMGs in newborn pigs, we were able to test for CF vs. non-CF differences prior to the onset of infection and inflammation (40). At that time point, the cell-type composition and transcripts did not differ by genotype. This result is consistent with previous mRNA microarray (69, 70). Lack of substantial differences between CF and non-CF SMG transcripts are consistent with the observation that early CF host-defense defects in antimicrobial activity and mucociliary clearance are reproduced simply by preventing Cl^−^ and HCO_3_^−^ secretion; these interventions that acidify the pH, decrease liquid secretion and increase protein concentration, functions mediated directly by CFTR anion channels (18, 20). In contrast, a Ca^2+^-activated Cl^−^ channel, *TMEM16A*, may have cell-intrinsic function, as *TMEM16A* gene disruption alters airway development and barrier function (71).

### Concluding Comments.

SMGs are essential for normal respiratory host defense in humans and large mammals (1, 8). These data enlarge our knowledge of these structures, identify their cellular and molecular composition, and provide a baseline for understanding how disease and environmental challenges alter the lungs.

## Methods

A more detailed version of methods is provided in *SI Appendix*.

### Human Donor Lungs.

Human lung tissues were obtained from the Iowa Donor Network through the In Vitro Models and Cell Culture Core of the University of Iowa. All studies were reviewed and approved by the Animal Care and Use Committee of the University of Iowa.

### Pigs and Porcine Tissue Harvest.

Wild-type and *CFTR*^−/−^ pigs (36) were generated and delivered by Exemplar Genetics. Newborn pigs for tissue harvest were 8 to 15 h after birth. They were anesthetized and killed. Tracheal segments were harvested and kept in cold 1× DPBS before further procedure.

### Tissue Dissociation and scRNA-Seq.

SMG and surrounding tissues were dissected from the trachea of newborn pigs (Dataset S7). Before dissection, airway surface epithelial cells were scraped off and the remaining epithelial layer was separated from the cartilage layer of the trachea to reveal SMGs. Dissected SMGs were successively dissociated with collagenase/hyaluronidase and pronase/DNase medium at 37 °C. After that, dissociation was terminated using cold 1× DPBS with fetal bovine serum. Cells were further washed with cold 1× DPBS and filtered with 40-µm tissue strainers. Single cells were spun down and resuspended in 1× DPBS with 0.4 mg/mL bovine serum albumin at around 1,000 cells/Ll. Single-cell droplets were generated using 10X Genomics Chromium Controller in the core facility of the Iowa Institute of Human Genetics of the University of Iowa. Next, 5,000 cells were targeted for single-cell droplets. cDNA libraries were generated using the Chromium Single Cell 3′ Reagent Kits v2 system. cDNA libraries passed all the quality controls that were recommended by 10X Genomics before sequencing. Sequencing was conducted on a HiSEq. 4000 system.

### Data Processing and Quality Control.

Raw sequencing FASTQ data were converted to matrix data (counts for genes) using the Cellranger 3 toolkit. An updated RefSeq Sscrofa11.1 genome annotation was used as the genome reference. Matrix data were subjected to ambient RNA correction using the SoupX R package (72), doublet filtering using the Scrublet Python package (73), and dead and low-quality cell filtering in Seurat (74).

### Seurat-Based Single-Cell Dataset Integration and Clustering.

Single-cell datasets were integrated and clustered using the Seurat 3.1.4 R package (74) following the typical integration tutorial (https://satijalab.org/seurat/v3.1/integration.html). In brief, matrix data were normalized at natural log scale using scale factor as 10,000. Two-thousand variable features were automatically selected for dataset integration. After integration, datasets were scaled regressing out “percent.mt” and cell-cycle identities, following principal component analysis with the first 30 principal components. Cell clusters were visualized in two-dimensional UMAP (75).

### Marker Gene Identification and Cluster Annotation.

Marker genes for each cluster were calculated using the function “FindAllMarkers” in Seurat. The Wilcoxon rank sum test was used. Testing was limited to genes that were expressed in more than 25% of a cell population and were differentially expressed on average at least 0.25-fold at natural log scale. Clusters were named based on specific marker genes that were previously reported for a certain cell-type in previous studies.

### Differential Expression Analysis.

Differential expression analysis between wild-type and *CFTR*^−/−^ was carried out using the function “FindMarkers” in Seurat. The Wilcoxon rank sum test was used, logfc.threshhold was set to 0.25.

### Immunostaining.

Formalin-fixed paraffin-embedded sections, cryosections, and whole tracheal epithelial tissues were processed following protocols described in *SI Appendix*, *Supplemental Methods*. Sections and tissues were preblocked with 10% serum before primary antibody incubation. The information of primary antibodies is listed in Dataset S8. After primary antibody incubation, sections and tissues were washed with 1× DPBS and incubated with Alexa Fluor secondary antibodies. Sections and tissues were mounted in antifade mounting medium (# H-1500, Vector laboratories) and imaged with confocal microscopy.

### smFISH.

An smFISH method named proximity ligation in situ hybridization was performed based on a published protocol (76) with a few modifications. In brief, tracheal segments were fixed in 4% formaldehyde, and 10-μm-thick cryosections were collected and preserved at −80 °C before in situ hybridization. On the day of in situ hybridization, tissue sections were postfixed in cold 4% PFA for 20 min. Sections were then incubated in prewarmed citrate solution with 0.05% lithium dodecyl sulfate and 0.05% Triton X-100 at 65 °C for 30 min. After that, sections were incubated with 0.05 mg/mL pepsin in 0.1N HCl for 10 min at 37 °C following 1× DPBS washing. At this stage, typical proximity ligation in situ hybridization was performed on hybridization-ready sections. The short mRNA sequences that were targeted by the paired hybridization probes are listed in Dataset S8.

### Transmission Electron Microscope Imaging.

Tracheal segments (<1 mm^3^) from newborn pigs were cut, briefly rinsed in cold 1× PBS, and fixed in glutaraldehyde fixation buffer (2.5% glutaraldehyde, 0.1 M sodium cacodylate). Tissues were next postfixed in osmium tetroxide (2%), and en-bloc stained with uranyl acetate (2.5%). Then, tissues were dehydrated through a graded ethanol series, infiltrated with Eponate 12, and polymerized at 60 °C for 24 h. Next, 70-nm-thick sections were cut and counterstained with 5% uranyl acetate and Reynold’s lead citrate. Images were taken using a transmission electron microscope (JEOL, JEM1230) with a Gatan UltraScan 2k × 2k CCD camera.

### Confocal Imaging.

Fluorescent images were taken using the Olympus FV3000 system. Individual field or multiple fields of *z*-stack images were scanned and stitched together. Images were processed to either single *z*-stack images or *z*-stack videos.

### Statistical Analysis.

Data points in graphs represent biological samples unless specifically indicated in the text. Results in graphs are presented as mean ± SD. Statistical significance was tested using Student’s *t* test, *P* values less than 0.05 were considered statistically significant.

## Supplementary Material

Supplementary File

Supplementary File

Supplementary File

Supplementary File

Supplementary File

Supplementary File

Supplementary File

Supplementary File

Supplementary File

Supplementary File

## Data Availability

The data reported in this paper have been deposited in the Gene Expression Omnibus (GEO) database, https://www.ncbi.nlm.nih.gov/geo (accession no. GSE185849) (77).
