# Supplementary material for: Cellular and molecular architecture of submucosal glands in wild-type and cystic fibrosis pigs
Source: Proc Natl Acad Sci U S A. 2022 Jan 19;119(4):e2119759119. doi: 10.1073/pnas.2119759119 (PMC8794846; doi:10.1073/pnas.2119759119)
Supplement: Supplementary File [file pnas.2119759119.sapp.pdf]

Supplementary Information for
Cellular and Molecular Architecture of Cells in Submucosal Glands of Wild-Type and Cystic Fibrosis Pigs

Wenjie Yu[1], Thomas O. Moninger[1], Andrew L. Thurman[1], Yuliang Xie[1], Akansha Jain[1], Keyan Zarei[1], Linda S. Powers[1], Alejandro A. Pezzulo[1], David A. Stoltz[1,2,3], Michael J. Welsh[1,2,4]

Departments of Internal Medicine[1] and Molecular Physiology and Biophysics[2],
Pappajohn Biomedical Institute,
Roy J. and Lucille A. Carver College of Medicine,
University of Iowa, Iowa City, Iowa 52242

Department of Biomedical Engineering[3],
University of Iowa, Iowa City, Iowa 52242

Howard Hughes Medical Institute[4]
University of Iowa, Iowa City, Iowa 52242

Address correspondence to: michael-welsh@uiowa.edu

This PDF file includes:

Supplementary Methods
Figures S1 to S14

Other supplementary materials for this manuscript include the following:

Movies S1
Datasets S1 to S8

SUPPLEMENTAL METHODS

Tracheal epithelial layer peeling and submucosal gland (SMG) exploring

The principle of this assay is that airway surface epithelium and SMGs are tightly connected, but their connection with smooth muscles and cartilage are loosened. Scissors and very fine tweezers (12-000-131, Fisher Scientific) were sterilized beforehand. Tracheal segments were washed with ice-cold 1x DPBS (#14190144, Life Technologies), then were cut open at the ventral side in dish with 1x DPBS. Airway epithelial layer is the first tissue layer from the luminal side with a higher light reflection ratio duo to its higher cell density. The epithelial layer was picked up by a tweezer and the cartilage layer was held using another tweezer. Next, the epithelial layer was gently pulled away and eventually separated from the cartilage layer. By doing so, a peeled epithelial layer included airway surface epithelium at one side and the acini of SMGs at the other side.

SMG dissection

The tracheal epithelial layer was peeled and separated following a typical procedure above. Tracheal surface epithelial cells were scraped off from the epithelial layer before peeling since surface epithelial cells would drop off and contaminate the later procedure. The peeled tissues were briefly washed in cold 1x DPBS with dithiothreitol (DTT, 2 mM). Then SMGs were dissected off from the whole epithelial layer under a dissecting microscope. SMG tissues were collected and centrifuged at 500 g in 1 x DPBS with DTT (2 mM). Lastly, SMG tissues were washed with cold ammonium chloride solution (# 07800, STEMCELL Technologies) for 5 min and 1 x DPBS, respectively.

Tissue dissociation and single-cell preparation

Dissected SMG tissues were spun down and resuspended with 1 x Collagenase/Hyaluronidase (# 07912, STEMCELL Technologies) in DMEM with 1% penicillin and streptomycin. Tissues were transferred into Corning ultra-low attachment cell culture flasks (# CLS3815-24EA, Millipore Sigma) and were dissociated for 30 minutes in a cell culture incubator. After that, tissues were spun down and resuspended in dissociation medium with Pronase (1.4 mg/ml) and DNase (50 U/ml). The tissues were dissociated for another 2 hours in a cell culture incubator. During the dissociation period, flasks with tissues were gently shaken every 10 minutes. Tissue dissociation was terminated by adding 1 volume of cold 1 x DPBS with 10% fetal bovine serum. Dissociated cells were filtered with 40-μm tissue strainers to collect single-cells. Then single cells were spun down (500 g, 5 minutes), washed with 1 x DPBS twice, and resuspended in 1 x DPBS with 0.4 mg/ml bovine serum albumin (# 74719, New England Biolabs) at around 1000 cells/μl. Cell viability was measured by trypan blue staining. Samples with 90% or higher cell viability were subjected for further processing.

Pig reference generation for scRNA-seq

We generated pig references with pig genome annotation files from both Ensembl (Sscrofa 11.1.95) and RefSeq (Sscrofa11.1) databases. We used the “mkref” function of the Cellranger 3.0.1 (10x Genomics) to generate the Ensembl reference directly. For RefSeq reference, we used the “gffread” function from cufflinks package (http://cole-trapnell-lab.github.io/cufflinks/) to converted the Sscrofa11.1.gff annotation file to Sscrofa.11.1.gtf annotation and then generated the reference. We only adapted the

annotation for exons of all genes since Cellranger only requires the annotation of exons as reference.

Converting raw FASTQ data to matrix datasets

We converted raw FASTQ data to matrix data using the "count" function of Cellranger. The quality of raw sequencing reads alignment was visualized in Integrative Genomics Viewer (http://software.broadinstitute.org/software/igv/) (1). We noticed that reads for many genes were not well aligned to the reference genes, most of which were due to the lack of 3' untranslated region of those reference genes.

Improving pig genome annotation

To get an overall view of the accuracy of the reference genome annotation, we manually checked sequencing reads alignment for more than 6,000 genes that are expressed in human, pig, or mouse airways (Dataset S6) in Integrative Genomics Viewer. We adapted Sscrofa11.1 from RefSeq database (https://www.ncbi.nlm.nih.gov/genome/annotation_euk/Sus_scrofa/106/) as the default reference because it provided a better alignment for mucin genes and secretory genes comparing to Ensembl database {http://useast.ensembl.org/Sus_scrofa/Info/Index}. Genes with apparent annotation issues (reads dropping off or gene body overlapping) were further checked in UCSC Genome Browser (http://genome.ucsc.edu/cgi-bin/hgGateway). The modification that we applied to the original RefSeq annotation includes: 1) Adding or elongating the 3' UTR; 2) Reducing the overlapping of two genes with the same direction at their UTR region; 3) Updating the gene names; 4) Adding new genes based on other pig genome database (for example, mitochondria genes from Ensembl) or existing genes in human and mice. In addition, all the modifications had to refer to existing annotations among UCSC, RefSeq, Ensembl, and Genbank mRNA databases.

Ambient RNA correction

Ambient RNA contamination was estimated and then filtered using the SoupX R package (https://github.com/constantAmateur/SoupX) (2). Raw matrix datasets generated from Cellranger were processed individually. First, each dataset was pre-clustered, and contamination genes were estimated using the function "setContaminationFraction". Several serous-cell markers that are highly expressed in SMGs showed up as contamination source. Next, contamination fractions were calculated using the function "calculateContaminationFraction" by setting "nonExpressedGeneList" to *OBP2B*, *DMBT1*, *BPIFA1*, *BPIFB1*, *VMO1*, which were specifically and highly expressed in the serous cells of SMGs. Last, ambient RNA counts were corrected and new matrix datasets were generated using the function "adjustCounts".

Quality control of single-cell datasets

Doublet scores for single-cells from individual datasets were calculated using the function "scrub.scrub_doublets" in the Scrublet python package (https://github.com/AllonKleinLab/scrublet) (3). Threshold of doublet scores was determined based on the "scrub.plot_histogram", which was set as 0.3 for SMG datasets.

Percentage of mitochondria genes in a single-cell was calculated using the function "PercentageFeatureSet" in Seurat R package (https://github.com/satijalab/seurat) (4).

Low quality cells were filtered using the following threshold: 1000 < nCount_RNA < 50000, 500 < nFeature_RNA < 6000, percent.mt < 15, doublet score < 0.3.

Seurat-based single-cell datasets integration and clustering
Single-cell datasets were integrated and clustered using Seurat 3.1.4 R package (4) following the typical integration tutorial (https://satijalab.org/seurat/v3.1/integration.html). The exact functions and settings were listed below:
NormalizeData: normalization.method = "LogNormalize", scale.factor = 10000;
FindVariableFeatures: selection.method = "vst", nfeatures = 2000;
CellCycleScoring: cell-cycle genes were adapted from (5), and were converted to the gene names of *Sus scrofa*;
SelectIntegrationFeatures: nfeatures = 2000;
FindIntegrationAnchors: normalization.method = "LogNormalize", dims = 1:40;
IntegrateData: dims = 1:40;
ScaleData: vars.to.regress = c("percent.mt", "S.Score", "G2M.Score");
RunPCA: npcs = 30; RunUMAP: dims = 1:30; FindNeighbors: dims = 1:30;
FindClusters: resolution = 0.2.

Cell type correlation coefficient analysis
Averaged gene expression for all cell types were calculated using the function "AverageExpression" in Seurat. Then the outputs were subjected to pearson correlation coefficient calculation using the function "cor" in R.

Calculation of single-cell gene set enrichment score
Gene set enrichment scores in single cells were calculated using the function "AddModuleScore" in Seurat R package. Enrichment scores (mean expression of gene sets) for cell types were then calculated using the "aggregate" function in R, and were scaled using the "scale" function in R.

Immunostaining
For immunostaining on formalin-fixed paraffin-embedded sections, tissues were fixed in 10% neutral buffered formalin for 24 hours on a shaker at room temperature. After fixation, tissues were embedded in paraffin following a typical dehydration, clearing, infiltration, and embedding protocol. Sections (4-5 µm) were cut, deparaffinized, rehydrated, and subjected for antigen retrieval using citric buffer (pH 6) in 100 °C water bath for 20 minutes. After treating with 1 x DPBS with 0.2% triton x-100 and serum-based blocking solution, sections were incubated with primary antibodies (Dataset S8) for 2 hours at room temperature or about 16 hours at 4 °C. Immunostaining signals were generated using either Alexa fluorescent dye labeled secondary antibodies or horseradish peroxidase and diaminobenzidine labeling kit (# DAB150, Millipore Sigma). Sections were mounted in mounting mediums and imaged in confocal microscopy or bright field microscopy.

For immunostaining on cryosections, tissues were fixed in 4% formaldehyde for 4-6 hours on a shaker at 4 °C. After fixation, tissues were dehydrated in 30% sucrose until sinking to the bottom of the medium, and then were embedded in optimal cutting temperature (OCT) compound using a flash frozen method. Cryosections (10 μm) were cut, washed with 1 x DPBS, permeabilized with 0.2% triton x-100 in 1 x DPBS, and blocked in 10% serum-based blocking solution. Sections were incubated with primary antibodies (Dataset S8) in 1 x DPBS with 0.1% tween 20 for 2 hours at room temperature or about 16 hours at 4 °C in a humidified box, followed by three times of washing and secondary antibody (Dataset S8) incubation. Sections were mounted in antifade mounting medium (# H-1500, Vector laboratories) and imaged with confocal microscopy.

For whole tracheal epithelial tissue immunofluorescent staining, tracheal epithelial layers with SMGs were fixed in 4% formaldehyde at 4 °C overnight. Tissues were washed with 1 x DPBS, permeabilized in 1 x DPBS with 0.3% triton x-100 for 6-8 hours at 4 °C, blocked with serum-based blocking solution (1 x DPBS, 10% serum, 0.2% triton x-100) at 4 °C overnight. Tissues were next sequentially incubated with primary antibodies at 4 °C for 24 hours, secondary antibodies at 4 °C for 4-6 hours, and 1μg/ml 4′,6-diamidino-2-phenylindole (DAPI) in 1 x DPBS at 4 °C for an hour. Tissues were mounted in antifade mounting medium, kept in 4 °C overnight, and imaged with confocal microscopy.

Single-molecule fluorescence *in situ* hybridization (smFISH)

A smFISH method named proximity ligation *in situ* hybridization was conducted based on the protocol shared by Dr. Nagendran and Dr. Desai from Stanford University (6). Tracheal segments were fixed in 4% formaldehyde for 24 hours on shaker at 4 °C. Tissues were dehydrated in cold 30% sucrose until sinking to the bottom of containers. Tissues were then embedded in OCT medium and sectioned at 10 μm sickness. Frozen tissue blocks and sections were stored at -80 °C for up to 3 months.

On the day for *in situ* hybridization, sections were fixed in ice-cold 4% formaldehyde for 20 minutes. Fixed sections were then dehydrated in 95% and 100% ethanol for a total time of 3 minutes and air dried for 10 minutes at room temperature. Sections were next incubated in prewarmed 1 x citrate solution (pH 6, H-3300, Vector laboratories) with 0.05% lithium dodecyl sulfate (L4632, Sigma) and 0.05% triton x-100 at 65°C for 30 min. After that, sections were rinsed with RNase-free water and air dried. A hydrophobic barrier was drawn around tissue sections using an immedge hydrophobic barrier pen. Next, sections were incubated with 0.05 mg/ml pepsin (P6887, Sigma-Aldrich) in 0.1N HCl for 10 min at 37 °C following one time of 1× DPBS washing. At this stage, sections were ready for oligonucleotide probe hybridization.

We applied the typical proximity ligation *in situ* hybridization protocol to hybridization-ready sections. In brief, sections were incubated with 2-5 short paired hybridization probes at 37 °C for 2 hours. Sections were then incubated with circulation probes at 37 °C for 1 hour, following with the ligation of circulation probes using T4 DNA ligase. Sections were next incubated with Phi29 polymerase (# 30221, Lucigen) for rolling-circle amplification at 37 °C for 4 hours. Lastly, sections were incubated with fluorescent-

labeled oligonucleotide and mounted with antifade mounting medium (# H-1500, Vector laboratories). The short mRNA (cDNA) sequences that were targeted by paired hybridization probes were listed in Dataset S8.

Tissue preparation and RNA extraction

Tracheal segments from newborn *WT* and *CF* pigs were incubated with pronase (1.4 mg/ml) and DNase (50 U/ml) in cell culture incubator for 2 hours. Dissociation was terminated by adding 1 volume cold 1x DPBS with 10% FBS. Single cells were collected after filtering the dissociation mix with 40 μm tissue strainer, which were considered as pure airway surface epithelium. The remaining tracheal segments were further washed with 1 x DPBS, and then epithelial layers were peeled off from the smooth muscle and cartilage parts, which were considered as SMG and surrounding tissue group. Tissues were stored in liquid nitrogen before RNA extraction. On the day of RNA extraction, 1-2 ml of TRIzol (Invitrogen) were added to freezing tissues, then tissues were mechanically disrupted. RNeasy kit (# 58730, Qiagen) was used to purify RNAs following the protocol from the kit.

Reverse transcription and quantitative PCR

High-Capacity cDNA Reverse Transcription Kits (# 4368814, Thermo Fisher Scientific) were used for reverse transcription. 2 μg RNAs from each sample were applied to 40 μl reaction mix with reverse transcriptase, random primers, and dNTPs. cDNAs were made following recommended thermal cycling conditions.

Fast SYBR Green Master Mix (# 4385612, Applied Biosystems-Life Technologies) was used for quantitative PCR. Tested genes were normalized to their cell-type specific reference genes (For example, *EPCAM* for all the genes that are expressed in epithelium) to generate ΔCT values. Fold changes were determined using ΔΔCT values. Primers for this assay were listed in Dataset S8.

REFERENCES FOR SUPPLEMENTAL METHODS

1. J. T. Robinson *et al.*, Integrative genomics viewer. *Nat Biotechnol* **29**, 24-26 (2011).
2. M. D. Young, S. Behjati, SoupX removes ambient RNA contamination from droplet-based single-cell RNA sequencing data. *Gigascience* **9** (2020).
3. S. L. Wolock, R. Lopez, A. M. Klein, Scrublet: computational identification of cell doublets in single-cell transcriptomic data. *Cell Syst* **8**, 281-291 e289 (2019).
4. T. Stuart *et al.*, Comprehensive integration of single-cell data. *Cell* **177**, 1888-1902 e1821 (2019).
5. I. Tirosh *et al.*, Dissecting the multicellular ecosystem of metastatic melanoma by single-cell RNA-seq. *Science* **352**, 189-196 (2016).
6. M. Nagendran, A. M. Andruska, P. B. Harbury, T. J. Desai, Advances in proximity ligation in situ hybridization (PLISH). *Bio Protoc* **10**, e3808 (2020).

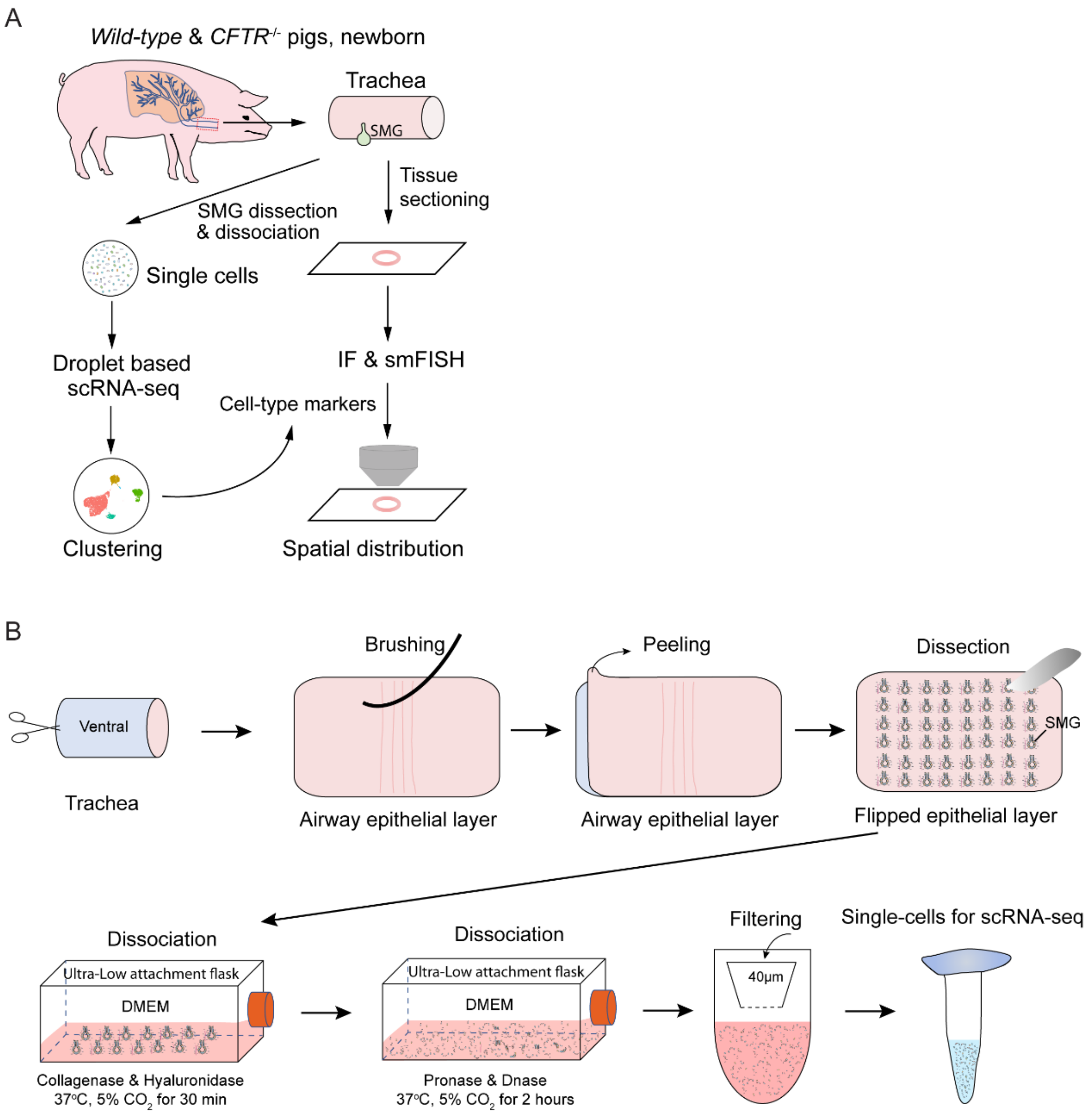

Figure S1. Schematic of single-cell RNA sequencing (scRNA-seq) in SMGs
A) Schematic of scRNA-seq and cell-type spatial distribution analysis in SMGs. Tracheal segments were harvested from newborn *wild-type* and *CFTR*$^{-/-}$ piglets.
B) Schematic of protocol for single cell preparation from newborn pig tracheal SMGs.

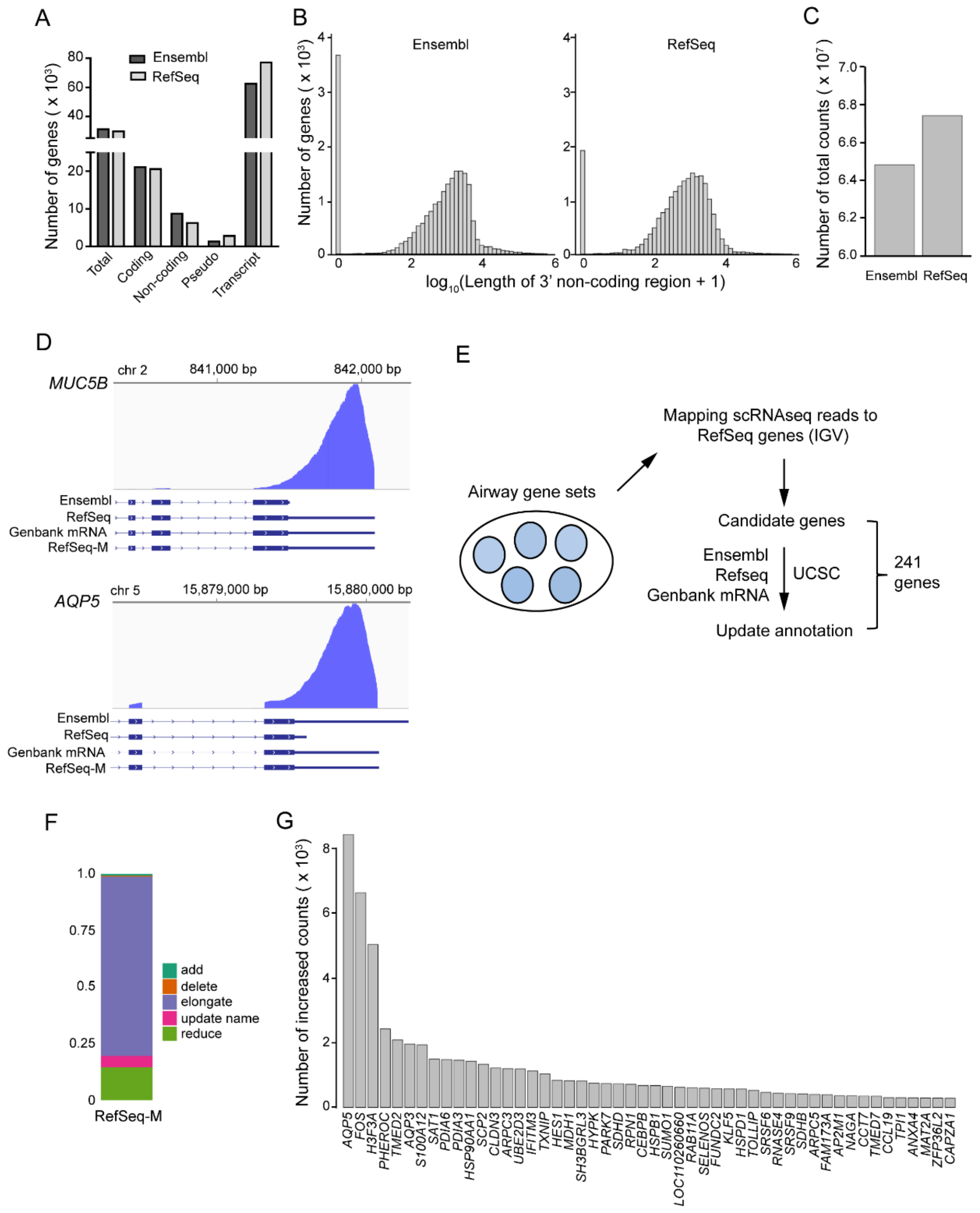

Figure S2. Pig reference modification.
A-B) Comparison of pig genome references from Ensembl and RefSeq databases.
C) Total counts of a scRNA-seq dataset using either Ensembl or RefSeq reference.
D) Raw sequencing reads of representative genes mapped to different annotation references. Ensembl, Ensembl database; RefSeq, RefSeq database; mRNA, GenBank mRNA database; RefSeq-M, improved RefSeq reference according to three databases above.

E-F) Summary of porcine reference modification. 241 genes were modified from the original annotation of Sscrofa11.1 from RefSeq database. Different types of modification are summarized in **F**.
G) Top 50 genes with the gain of counts from a SMG sample using updated RefSeq annotation.

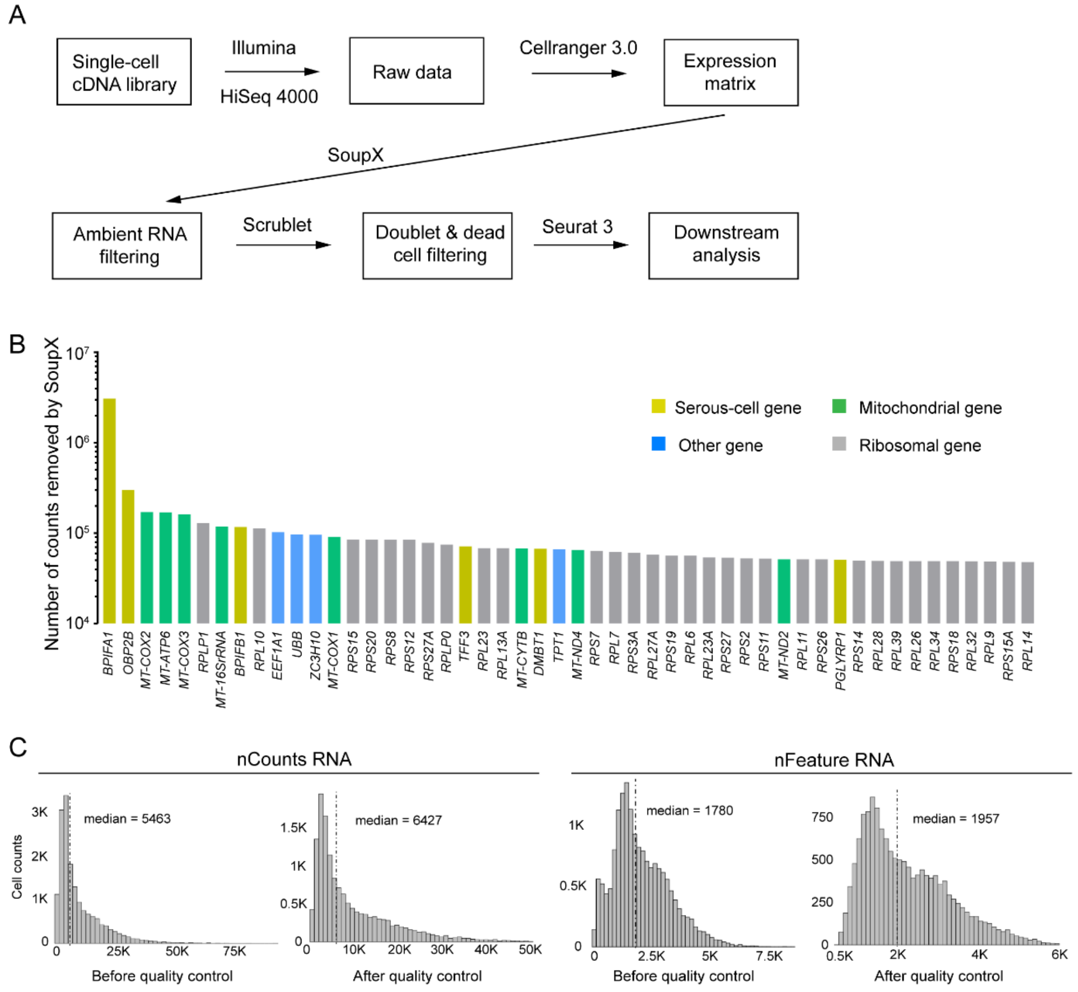

Figure S3. Quality control of scRNA-seq datasets.
A) Workflow of scRNA-seq dataset processing. Single-cell cDNA libraries were sequenced using HiSeq 4000 from Illumina. Raw sequencing datasets were then processed by Cellranger 3.0 toolkit from 10x Genomics. Expression matrix files were analyzed using SoupX R toolkit to filter ambient RNA counts. Cells in the expression matrix files were next filtered for potential doublet (Scrublet R toolkit) and low-quality identities (low number of gene & RNA counts, high proportion of mitochondrial gene counts). Lastly, filtered matrix files were processed with Seurat 3 R toolkit following typical online tutorials (https://satijalab.org/seurat)
B) Summary of filtered ambient transcripts from raw expression matrix in the entire scRNA-seq dataset using SoupX.
C) Quality metrics for scRNA-seq data before and after quality controls. Left panel, distributions of the number of transcripts (nCounts) per cell before and after quality control; Right panel, distributions of the number of genes (nFeature) per cell before and after quality controls.

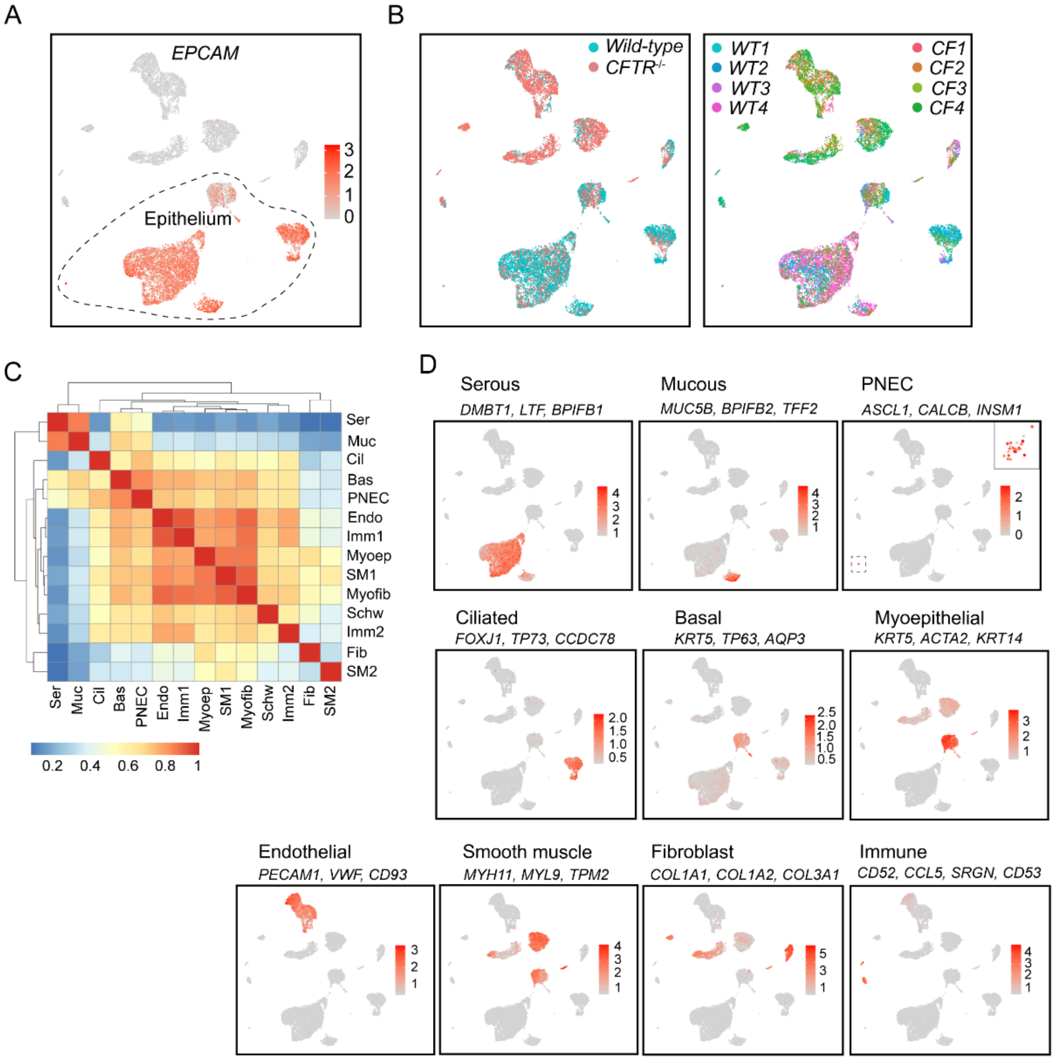

Figure S4. Summary of clusters in scRNA-seq data.
A) *EPCAM* expression visualized in UMAP. Epithelial clusters (*EPCAM*+) are outlined by dashed line.
B) UMAP of scRNA-seq data grouped by genotype (left) and individual samples (right).
C) Pearson correlation coefficient of all cell types.
D) Cell types defined by classic markers. Mean expression value of each marker-gene set was calculated and plotted in UMAP.

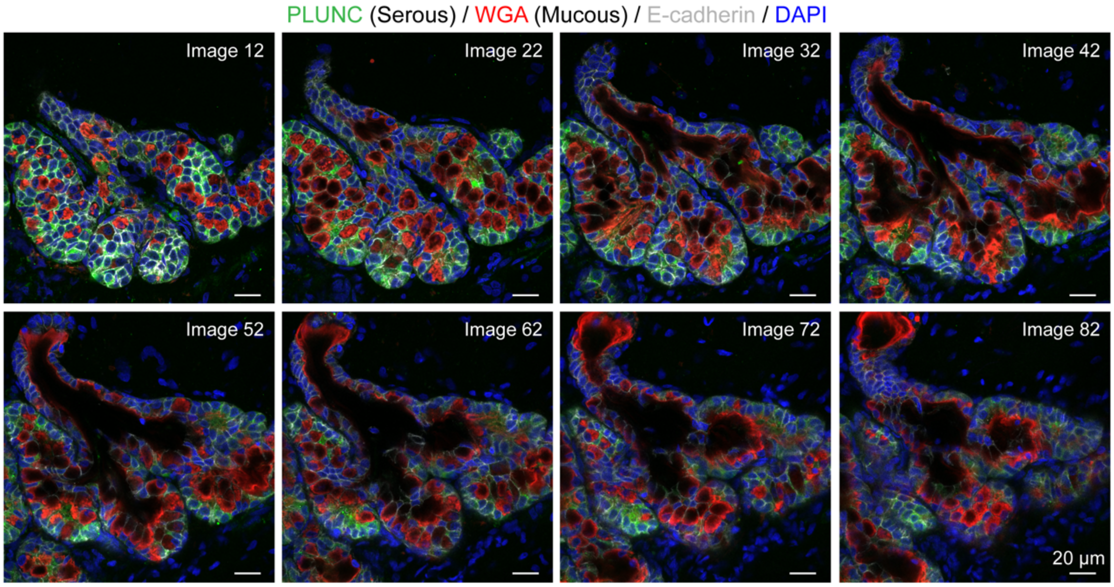

Figure S5.
Representative images from a stack of confocal images of a single SMG stained with PLUNC (green, a serous cell marker), WGA (a mucous cell marker), E-cadherin (a epithelial marker), and DAPI (a nuclear marker). For all images see Video S1.

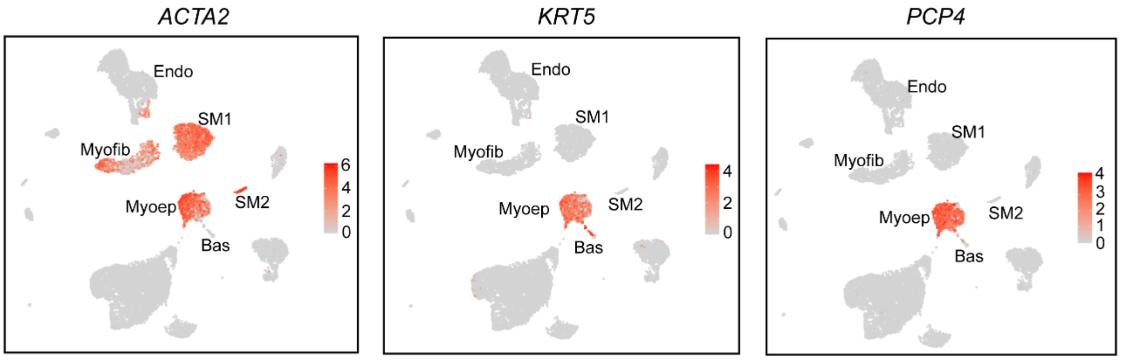

Figure 6.
Expression pattern of *ACTA2*, *KRT5*, and *PCP4* presented in the UMAP of scRNA-seq data. *PCP4* is limited to the myoepithelial-cell (Myoep) cluster.

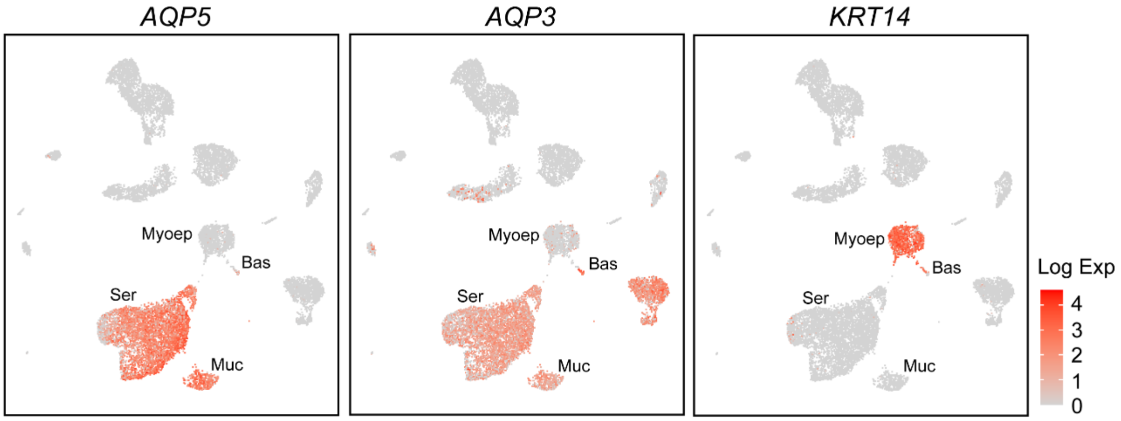

Figure S7.
Expression pattern of *AQP5*, *AQP3*, and *KRT14* presented in the UMAP of scRNA-seq data

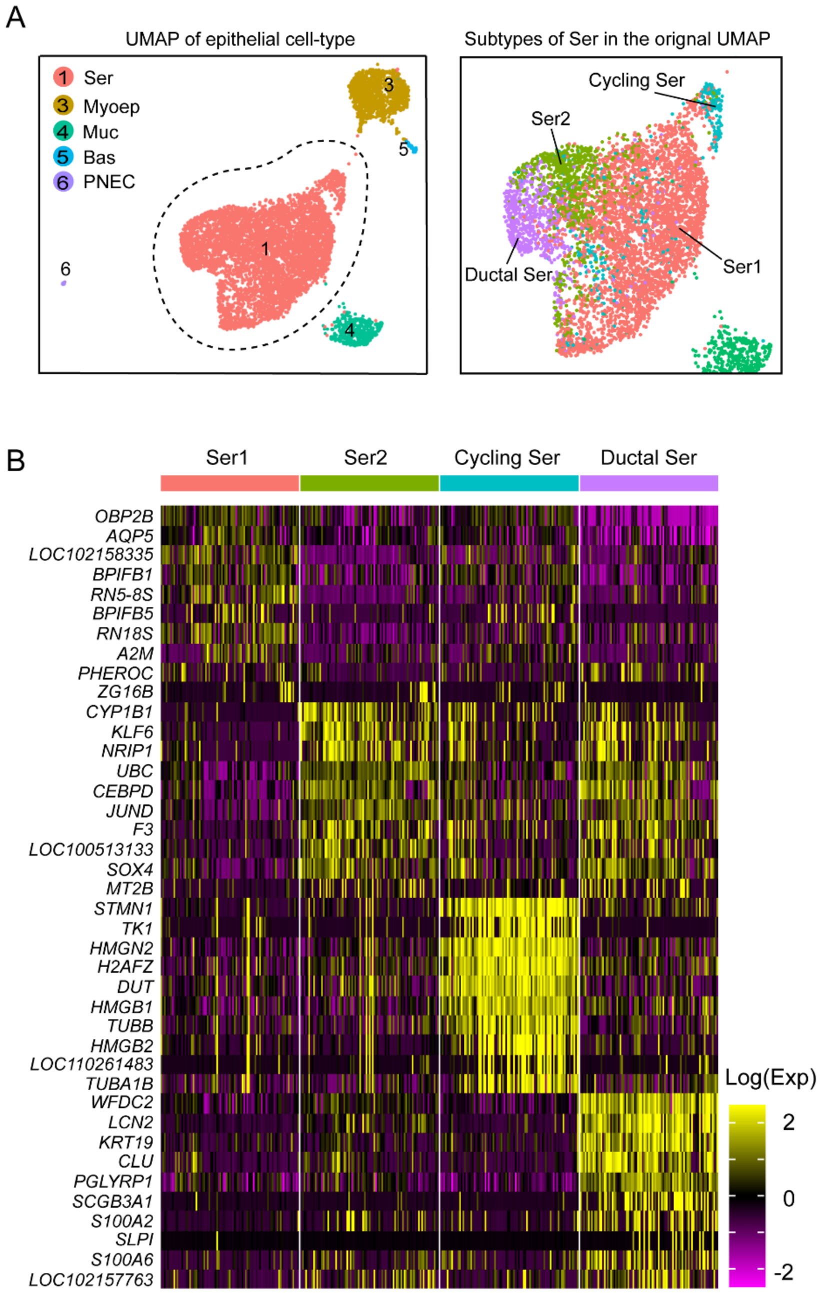

Figure S8.
A) Left, UMAP of all epithelial cell types in scRNA-seq data. Right, sub-clusters of serous-cell cluster in the original UMAP. Ser, serous cell; Cil, ciliated cell; Muc, mucous cell; Bas; basal cell; PNEC, pulmonary neuroendocrine cell; myoep, myoepithelial.
B) Heatmap of top 10 markers for subtypes of serous cells.

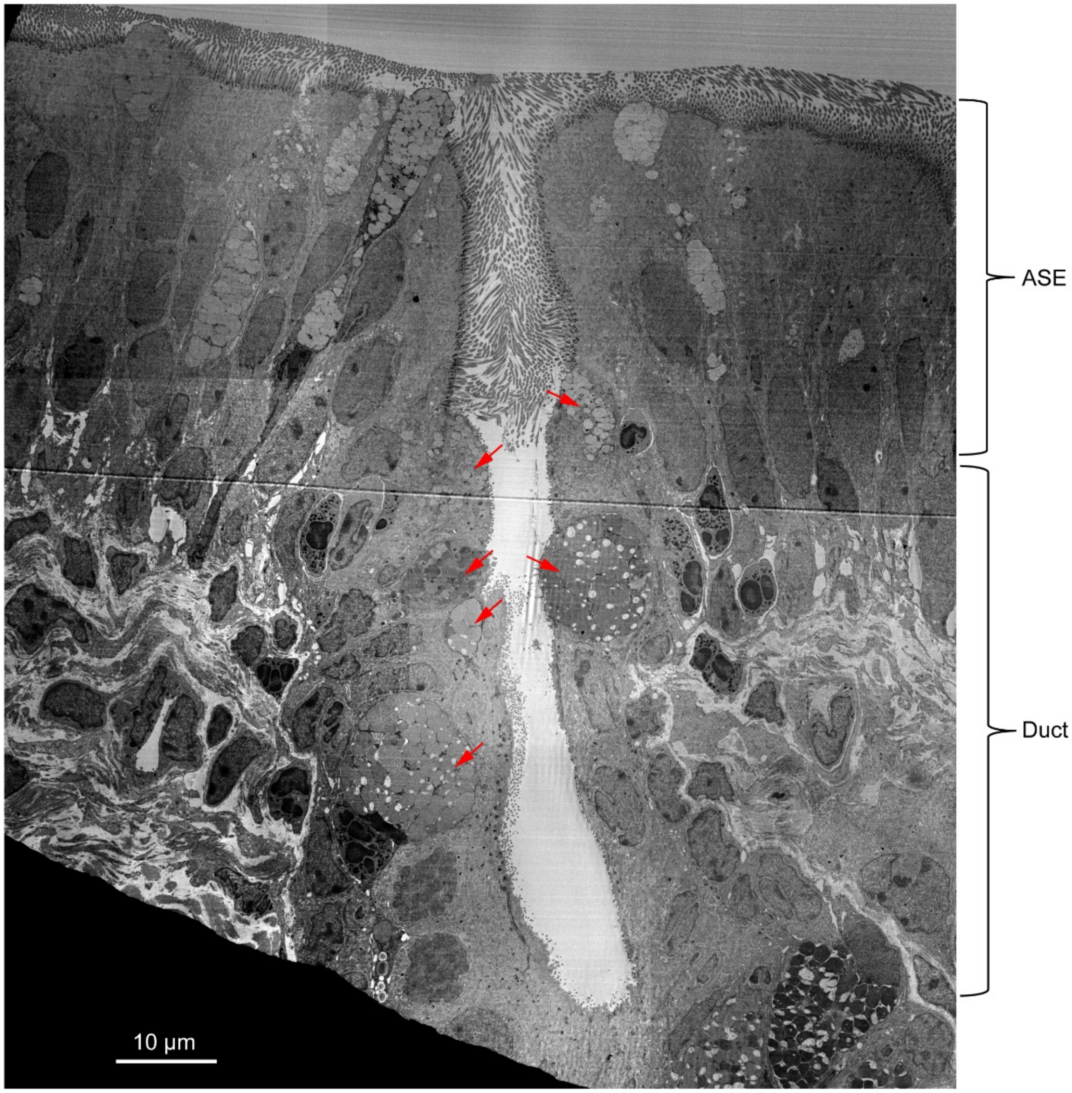

Figure S9.
Representative transmission electron microscopy image of SMG duct in newborn pig trachea. Arrows point to secretory vesicles in ductal serous cells. ASE, airway surface epithelia.

KRT5 / DAPI

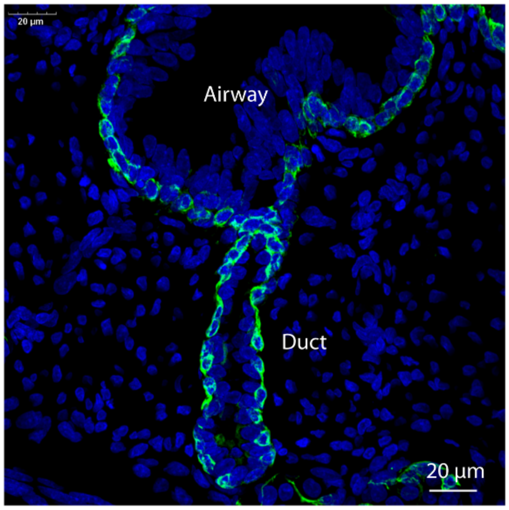

Figure S10.
Representative image of KRT5 (basal-cell marker) staining in tracheal sections. KRT5 only labels the cells at the out layer of SMG duct.

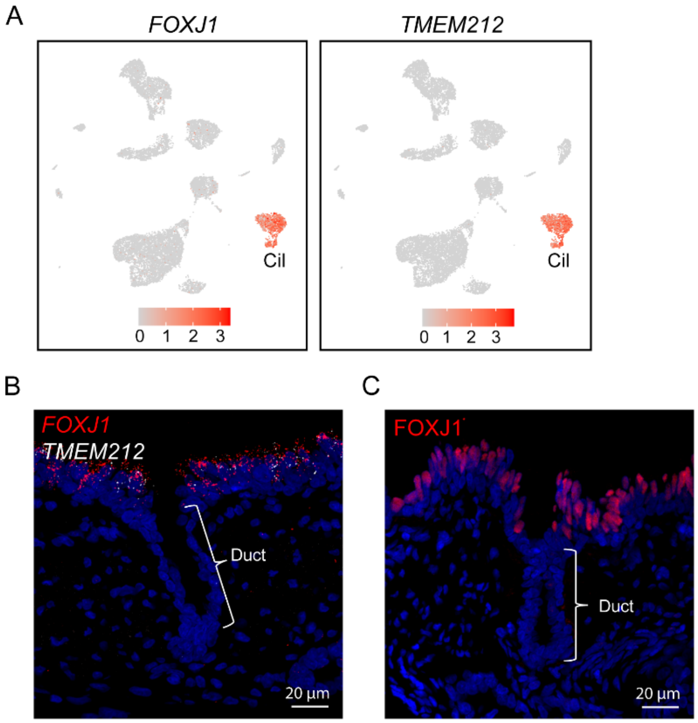

Figure S11. Distribution of ciliated cells in pig trachea.
A) Expression pattern of *FOXJ1* and *TMEM212* presented in the UMAP of scRNA-seq data. *FOXJ1* and *TMEM212* were specifically enriched in ciliated cell (Cil) cluster.
B, C) Representative images of smFISH and immunostaining of ciliated cell markers (FOXJ1, TMEM212) in airway surface and SMGs. SMG duct is defined as the ductal region beneath the airway surface epithelial layer.

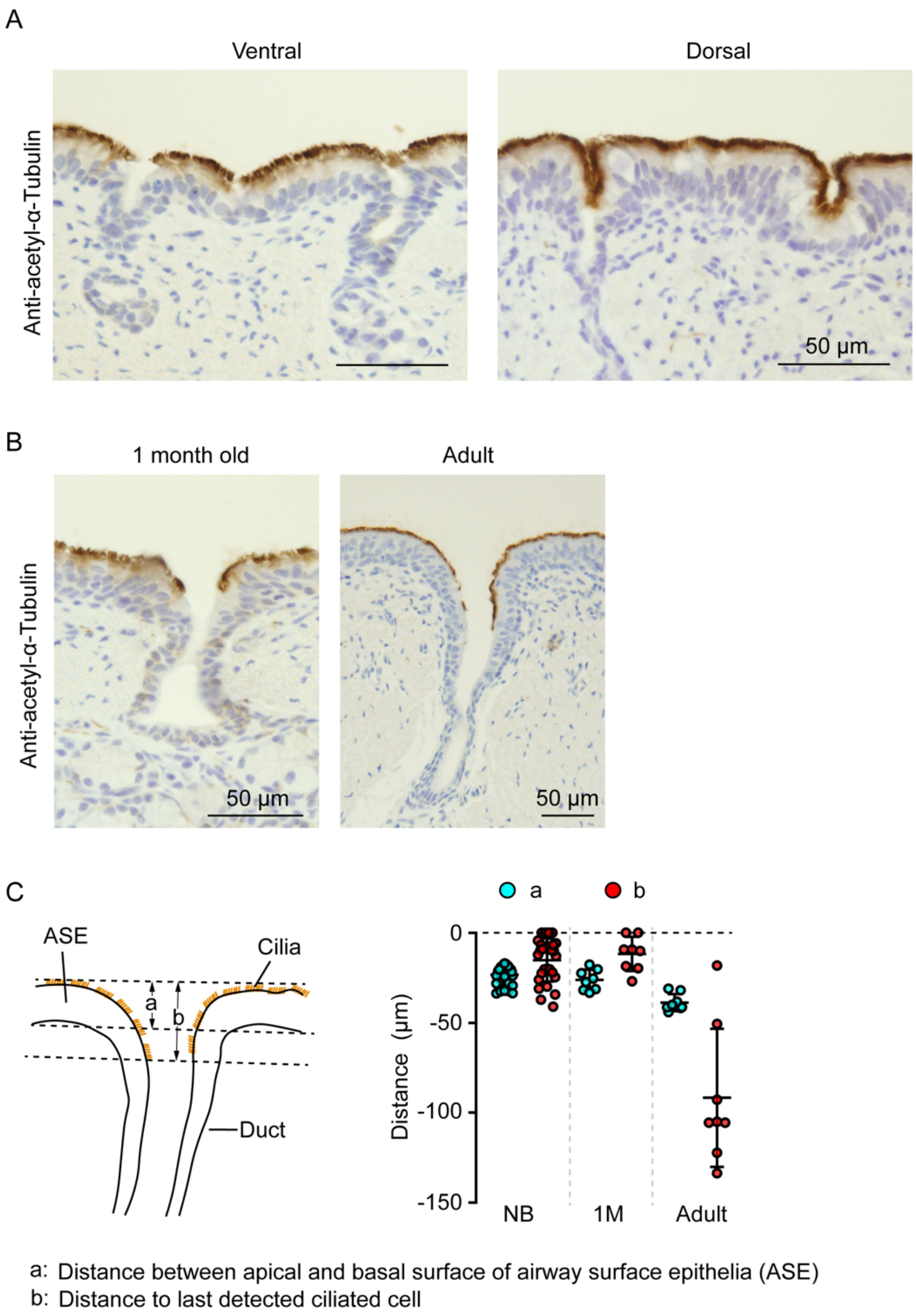

Figure S12. Immunostaining of ciliated cell marker acetyl-α-tubulin in airway sections.

A) Representative images of ciliary labeling in newborn pig tracheal sections.

B) Representative images of ciliary labeling in one-month old (left) and adult (right) pig tracheal sections. Ciliated cells do not extend beyond pseudostratified airway surface epithelial layer in postnatal pig trachea.

C) Diagram shows measurements of the thickness of airway surface epithelium and distance from apical surface to last ciliated cell in SMG duct. Graph on right shows measurements in SMG ducts across ages. Serial tracheal sections with acetyl-α–tubulin staining were imaged. Each dot represents results from a sagitally sectioned SMG duct.

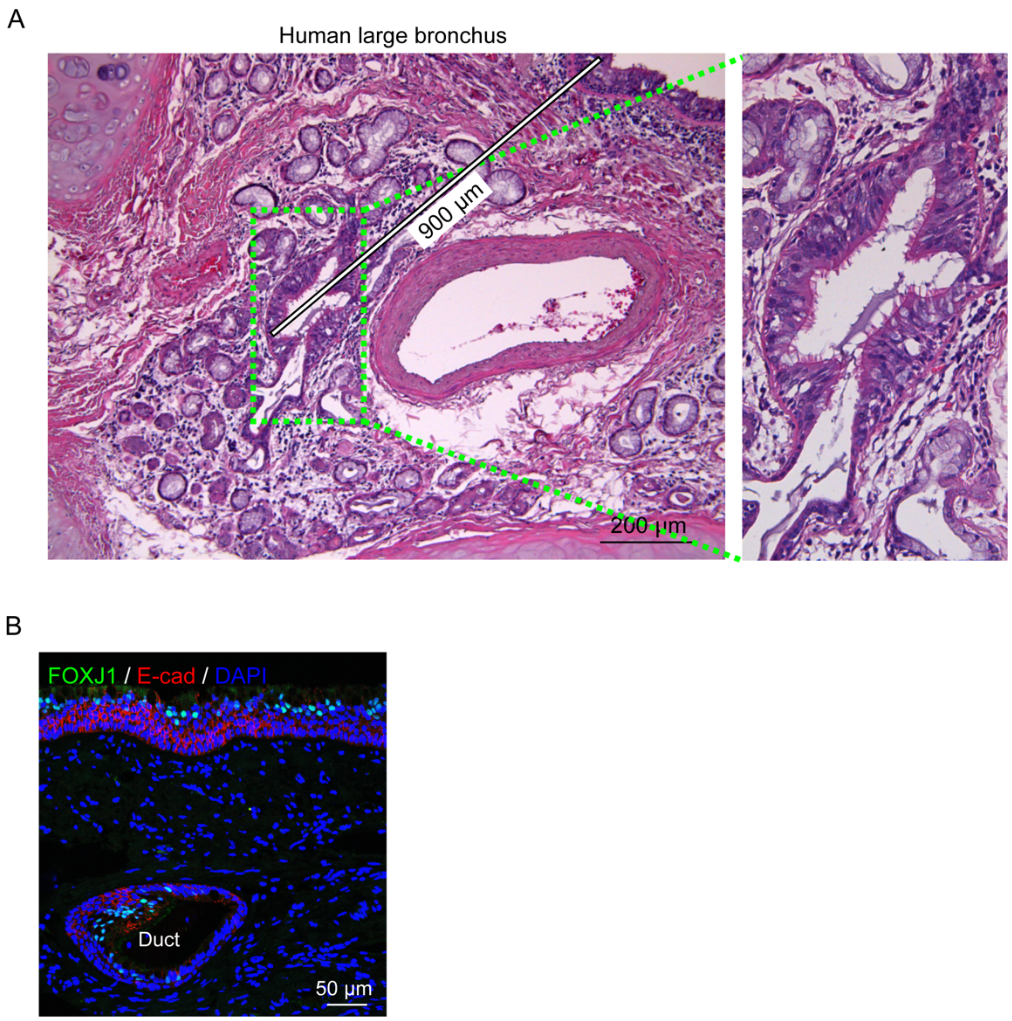

Figure S13. Distribution of ciliated cells in human SMGs.
A) Representative images of H&E staining of large human bronchi. Ciliated ducts were found deep (~ 1 mm) in submucosal region of airways.
B) Representative image of FOXJ1 expression in human airway surface and SMG ducts. Light blue (overlap of blue and green) indicates nuclear localization of FOXJ1.

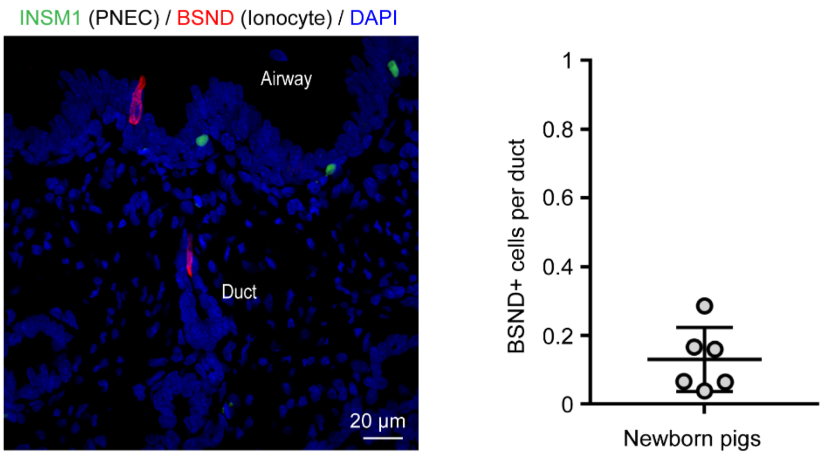

Figure S14.
Representative image of immunostaining of BSND (ionocyte marker) and INSM1 (PNEC marker) in airway surface epithelia and SMG duct. Graph on right shows number of BSND+ cells in SMG ducts. 5-6 serial tracheal sections were stained with anti-BSND antibody. Whole sections were imaged, stitched, and reconstructed together. The number of SMG ducts and ductal BSND+ cells were counted. Each dot represents a pig.
